# Compensation of Oxygen Transmittance Effects for Proximal Sensing Retrieval of Canopy–Leaving Sun-Induced Chlorophyll Fluorescence

**DOI:** 10.3390/rs10101551

**Published:** 2018-09-26

**Authors:** Neus Sabater, Jorge Vicent, Luis Alonso, Jochem Verrelst, Elizabeth M. Middleton, Albert Porcar-Castell, José Moreno

**Affiliations:** 1 Image Processing Laboratory (IPL), Parc Científic, Universitat de València, 46980 Paterna, València, Spain; 2 NASA, Goddard Space Flight Centre (GSFC), Greenbelt, MD 20771, USA; 3 Optics of Photosynthesis Laboratory, Institute for Atmospheric and Earth System Research (INAR/Forest Sciences), Department of Forest Sciences, University of Helsinki, P.O. Box 27, 00014 Helsinki, Finland

**Keywords:** sun–induced chlorophyll fluorescence (SIF), proximal sensing, O_2_ transmittance, fraunhofer line discriminator (FLD), spectral fitting method (SFM), air temperature, atmospheric pressure

## Abstract

Estimates of Sun–Induced vegetation chlorophyll Fluorescence (SIF) using remote sensing techniques are commonly determined by exploiting solar and/or telluric absorption features. When SIF is retrieved in the strong oxygen (O_2_) absorption features, atmospheric effects must always be compensated. Whereas correction of atmospheric effects is a standard airborne or satellite data processing step, there is no consensus regarding whether it is required for SIF proximal–sensing measurements nor what is the best strategy to be followed. Thus, by using simulated data, this work provides a comprehensive analysis about how atmospheric effects impact SIF estimations on proximal sensing, regarding: (1) the sensor height above the vegetated canopy; (2) the SIF retrieval technique used, e.g., Fraunhofer Line Discriminator (FLD) family or Spectral Fitting Methods (SFM); and (3) the instrument’s spectral resolution. We demonstrate that for proximal–sensing scenarios compensating for atmospheric effects by simply introducing the O_2_ transmittance function into the FLD or SFM formulations improves SIF estimations. However, these simplistic corrections still lead to inaccurate SIF estimations due to the multiplication of spectrally convolved atmospheric transfer functions with absorption features. Consequently, a more rigorous oxygen compensation strategy is proposed and assessed by following a classic airborne atmospheric correction scheme adapted to proximal sensing. This approach allows compensating for the O_2_ absorption effects and, at the same time, convolving the high spectral resolution data according to the corresponding Instrumental Spectral Response Function (ISRF) through the use of an atmospheric radiative transfer model. Finally, due to the key role of O_2_ absorption on the evaluated proximal–sensing SIF retrieval strategies, its dependency on surface pressure (p) and air temperature (T) was also assessed. As an example, we combined simulated spectral data with p and T measurements obtained for a one–year period in the Hyytiälä Forestry Field Station in Finland. Of importance hereby is that seasonal dynamics in terms of *T* and *p*, if not appropriately considered as part of the retrieval strategy, can result in erroneous SIF seasonal trends that mimic those of known dynamics for temperature–dependent physiological responses of vegetation.

## 1 Introduction

Sun–Induced chlorophyll Fluorescence (SIF) measured from remote sensing platforms provides a new optical means to track photosynthesis and gross primary production (GPP) of terrestrial ecosystems [1]. SIF consists of photons of red and near infrared (NIR) light that are emitted by chlorophyll (Chl) pigments as part of the de–excitation mechanisms in response to absorption of Photosynthetically Active Radiation (PAR). Since plants respond actively and continuously to different environmental conditions, continuous and long term observations become crucial in vegetation monitoring to understand terrestrial biosphere processes [2]. With this aim, in recent years many ground–based field spectroscopy systems have been developed and mounted on towers [3–5]. In addition, with production of global SIF maps from atmospheric satellites, e.g., [6–8], and with the forthcoming new ESA mission, the FLuorescence EXplorer (FLEX) [9], the use of tower–based SIF continuous measurements will play a key role in supporting validation and calibration activities for satellite and airborne measurements.

Many of the currently existing systems mounted on towers dedicated to detecting SIF typically measure both (1) the up–welling radiance coming from the surface, and (2) the incoming down–welling solar irradiance; at sensor level for practical reasons. The height of towers for measuring SIF mainly depends on the vegetation type to be monitored, ranging from 5–25 m to measure agricultural fields, to 20–50 m to monitor SIF over forests. Examples include: (1) the NASA FUSION tower (ftp://fusionftp.gsfc.nasa.gov/FUSION); (2) the TriFLEX instrument [10]; (3) the UNEDI system [11] at the FluxNet Hyytiälä site (http://fluxnet.ornl.gov); and two systems developed by Università degli Studi Milano–Bicocca: (4) the Multiplexer Radiometer Irradiometer (MRI) [12,13] and (5) the HyperSpectral Irradiometer (HSI) [14–16].

Although novel statistical techniques were recently developed to estimate SIF at both satellite and at proximal–sensing scales [17,18], when measuring SIF at Top Of the Canopy (TOC) the two state–of–the–art ground–based family techniques still relied on are based on the Fraunhofer Line Discriminator (FLD) principle [19–22] or on the Spectral Fitting Methods (SFM) [23–25]. Both FLD and SFM have been extensively used to measure SIF at the oxygen absorption features [26–31]. Notice that even though the FLD family of techniques was originally formulated to measure the SIF in–filling in the Fraunhofer solar lines [20,32], they are now commonly used for measurements in the terrestrial O_2_ bands (see references above). In particular, the O_2_–A band becomes advantageous when measuring SIF close to the target (~cm) since it provides a higher fractional depth than the Fraunhofer bands while neglecting atmospheric effects [17].

Despite this advantage, when increasing the optical path between the target and the sensor, if atmospheric effects are not corrected in the oxygen absorption regions, the estimated SIF can rapidly become prone to errors [33]. Because of that, past field campaign experiments dedicated to measuring SIF from hundreds of meters using airborne platforms always counted on atmospheric correction strategies as part of their retrieval processing [29,33–35]. Conversely, for instruments mounted on towers, the need to correct radiances acquired at the oxygen regions for atmospheric effects when aiming to estimate SIF is not so obvious. Notice that FLD and SFM families of SIF retrievals have been formulated to be applied at TOC level, assuming that: (a) the atmospheric path between the target and the sensor is short enough to be neglected; and (b) the solar irradiance is acquired directly at the same height as the target. However, these assumptions are not met when the atmospheric path length increases, as with a higher mount on towers or with larger solar or view zenith angles (SZA, VZA) [36]. Besides that, since oxygen absorption is proportional to air pressure [37], at the bottom of the atmosphere where pressure is greatest even a few meters distance between the target and the sensor can result in significant errors in the retrieved SIF at TOC [36].

It is worth mentioning that some field campaigns measuring SIF from systems mounted on towers have already incorporated a processing strategy to correct radiances for atmospheric effects [10,38,39]. Also, in a recent publication from X. Liu et al. (2017) [40], transmittance effects on SIF canopy–leaving measurements made with hemispherical sensors were analysed through an effective transmittance correction. Nevertheless, a comprehensive analysis that evaluates atmospheric effects on SIF measurements acquired at proximal sensing is still required. Therefore, to complement previous analyses about this topic under a theoretical framework, this paper focuses on assessing:

- How ignoring atmospheric effects can distinctly impact the success of the technique applied (FLD or SFM) to disentangle SIF from reflected light?
- What could be the best strategy to correct proximal sensing data for atmospheric effects?
- Is it possible to adapt atmospheric correction strategies used to process airborne data for proximal sensing measurements?

Additionally, this paper describes for the first time how to consistently couple formulations applied to correct proximal–sensing data for atmospheric effects with formulations used for retrieving SIF at canopy level. Our approach is similar to that in a previous study developed by Sabater et al. [41] where the coupling between atmospheric and SIF retrieval strategies was addressed at satellite scale. Both FLD and SFM methods are based on the idea that radiance measured at TOC, *L_TOC_*, is composed of the contributions of both the reflected solar irradiance and the emitted SIF. However, this condition is not exactly met when *L_TOC_* is not measured at TOC but, rather, estimated through inversion of an atmospheric correction scheme [41]. Radiance acquired at a certain distance from the target, *L_sen_*, is provided according to its specific Instrumental Spectral Response Function (ISRF), noted here as 〈*L_sen_*〉. Even in the most simple scenario when correcting the surface radiance acquired at the top (or some position) on a tower 〈*L_sen_*〉 for the atmospheric transmittance 〈*T*〉, this would imply dividing two non–smooth functions already convolved to derive 〈*L_TOC_*〉. Mathematically, given two functions *a* and *b*, this inequality always exists 〈*a*〉 · 〈*b*〉 ≠ 〈*a* · *b*〉, which is especially important when working with high spectral resolution data in absorption regions [41]. At the same time, it must be considered that atmospheric correction strategies generally assume simplifications in their formulation, e.g., assuming a Lambertian surface reflectance behaviour; or they must deal with the correction of adjacent contributions of light from surrounding areas.

Further, when focusing on the oxygen absorption spectral regions, O_2_ transmittance dependency on air temperature (T) and surface pressure (p) must be considered. In this paper, we aimed to assess to what extent seasonal changes in T and atmospheric p would interfere with the retrieved SIF signals, especially over long temporal data series when this dependency is not accounted for as part of the oxygen transmittance correction. In other words, the important question raised here is this: Can seasonal changes in T and p generate a significant false SIF signal that, if not appropriately compensated, mimics seasonal patterns related to temperature–dependent physiological responses expressed with vegetation SIF?

Altogether, this work aims to address all of these issues within a theoretical framework by using simulated data with the MODerate resolution atmospheric TRANsmission (MODTRAN) [42] atmospheric Radiative Transfer Model (RTM). The remainder of this paper is organized as follows. Section 2 describes the relationship between the measured at–sensor and at–surface solar irradiance and up–welling canopy radiances. Section 3 briefly reviews the FLD–family and SFM SIF retrieval approaches and modifies these standard formulations to introduce a first order O_2_ transmittance correction and analyses their limitations. We then propose to adapt a classical airborne atmospheric correction scheme for proximal sensing data, thereby compensating for O_2_ effects while also accounting for effects due to the instrument’s spectral resolution through the use of a radiative transfer model. Section 4 is dedicated to the results: (1) supporting the need to compensate for O_2_ absorption when retrieving SIF in proximal sensing; (2) reporting the expected SIF accuracy when a first order O_2_ compensation is applied to either the FLD or SFM retrievals for different instrument resolutions and sensor heights; and (3) reporting the expected SIF accuracy when a classical airborne atmospheric correction scheme is applied to compensate for the O_2_ transmittance. Section 5 examines the O_2_ transmittance dependency on T and p conditions and its potential impact when monitoring long temporal series. Section 6 discusses the main results found in this paper in a broader scientific context in view of experimental field campaigns and satellite validation activities. Finally, our main conclusions are summarized in Section 7.

## 2 Atmospheric Oxygen Transmittance Effects at Tower Scale

This section presents the theoretical background that relates the down–welling solar irradiance and the up–welling canopy radiance measured at different height configurations with different instruments. We quantified the expected O_2_ absorption when measuring the up–welling canopy radiance and the down–welling solar irradiance from tower instruments separated by distances of 0–20 m. In addition, mathematical aspects that are of importance when inverting high spectral resolution radiance spectra acquired at sensor level as part of the atmospheric correction process are also detailed.

### 2.1 At–Sensor and At–Canopy Solar Irradiance

For practical reasons, solar irradiance is commonly measured at a distance from the vegetated target location. Therefore, the relationship between the solar irradiance (*E*) measured at sensor level (e.g., on a tower or UAV), and the solar irradiance reaching the surface at TOC (*Ē*) is driven by Equation (1):

(1) $$\bar{E}∼E\cdot t^{↓}$$

where *t*^↓^ is the downward total (direct and diffuse) transmittance from the sensor level to the TOC level, for a given illumination geometry. Note that hereafter the over–line symbol on *Ē* (or over L) refers to magnitudes measured at the surface/canopy level. For the sake of readability, the spectral dependency of each term is omitted in the formulation presented in this section.

**Figure 1:**
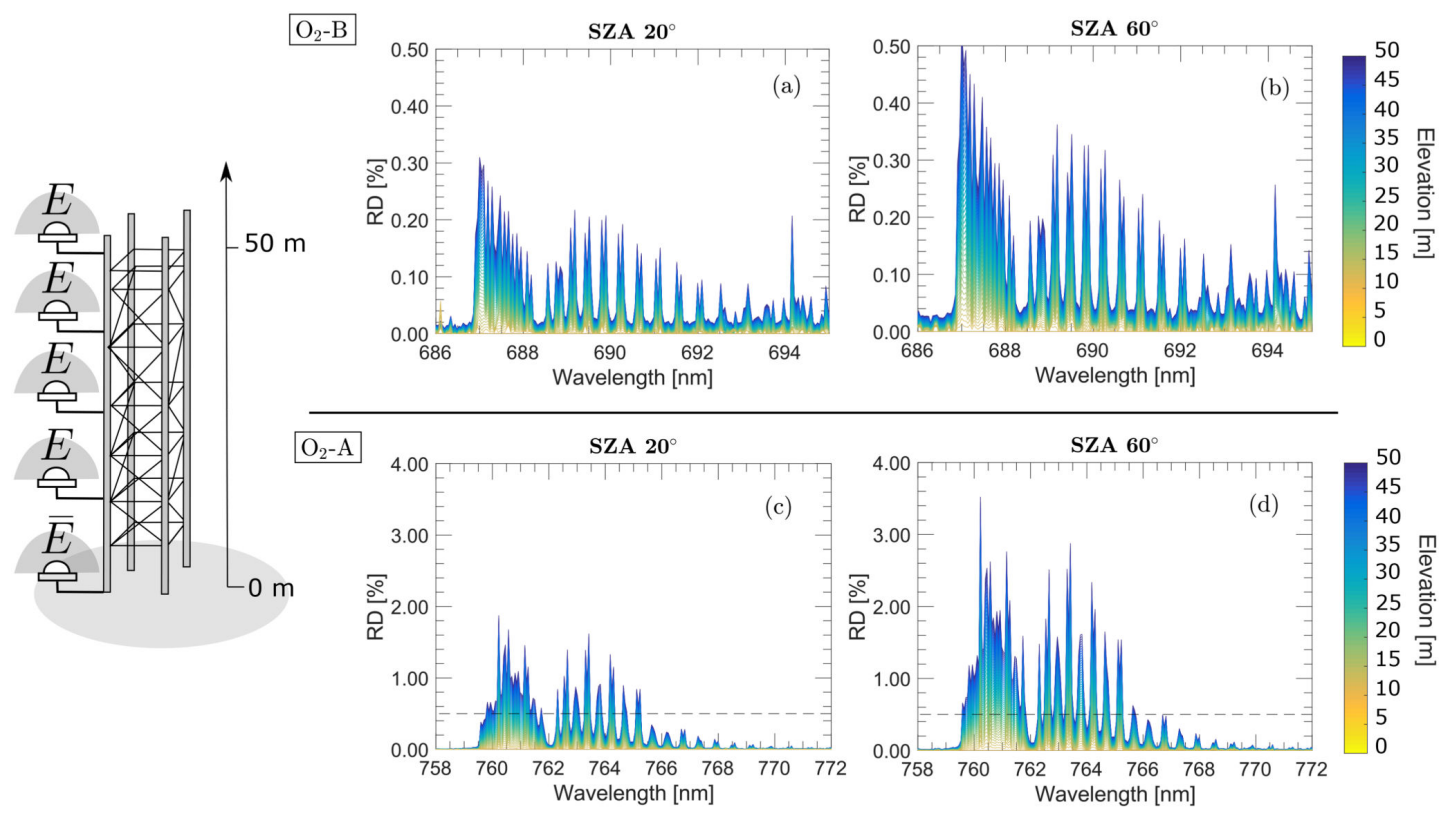
The percent (%) relative difference (RD) between the at–sensor (E) and at–surface (E) measured total solar irradiance for two different SZAs (20° and 60°) for a range of target–sensor distances between 0 to 50 m. Panels (**a**–**d**); show the solar irradiance RD (%) corresponding to the O_2_–B and O_2_–A regions, respectively. The black dashed–line in the panels (**c**,**d**) indicate the maximum RD reached in the O_2_–B region to facilitate the comparison. Note the difference in the RD(%) scales in (**a**,**b**) versus (**c**,**d**).

Accordingly to Equation (1), the difference between solar irradiance measured by the tower–mounted calibration panel (or sensor) and the actual solar irradiance reaching the surface at TOC will increase as either the distance between them, or the SZA, increase. Making use of MODTRAN, Figure 1 shows the relative difference in per cent (RD%) of the total solar irradiance, E, acquired at different sensor elevations for the O_2_–B and O_2_–A bands, and covering a range in SZAs. Details about inputs configured in MODTRAN are described in Table A1 of Appendix A.

### 2.2 Upward Atmospheric Transmittance from Surface TOC to Sensor Level

Assuming a Lambertian surface reflectance, the Top Of Atmosphere (TOA) radiance, *L_TOA_*, can be written as Equation (2):

(2) $$L_{TOA}=L_{0}+\frac{(\bar{E}\cdot \rho +\pi \cdot F)\cdot t^{↑}}{\pi \cdot (1-S\cdot \rho )}$$

where L_0_ is the atmospheric path radiance, *S* is the spherical albedo, *t*^↓^ is the total upward atmospheric transmittance (diffuse and direct contributions) from surface TOC to TOA, $\bar{E}$ is the solar irradiance reaching the surface, *F* is the emitted SIF (*F* hereafter for brevity) and *ρ* the Lambertian surface reflectance. Adjacency effects are not considered in Equation (2), and we are assuming an infinite uniform target with surface reflectance, *ρ*.

**Figure 2:**
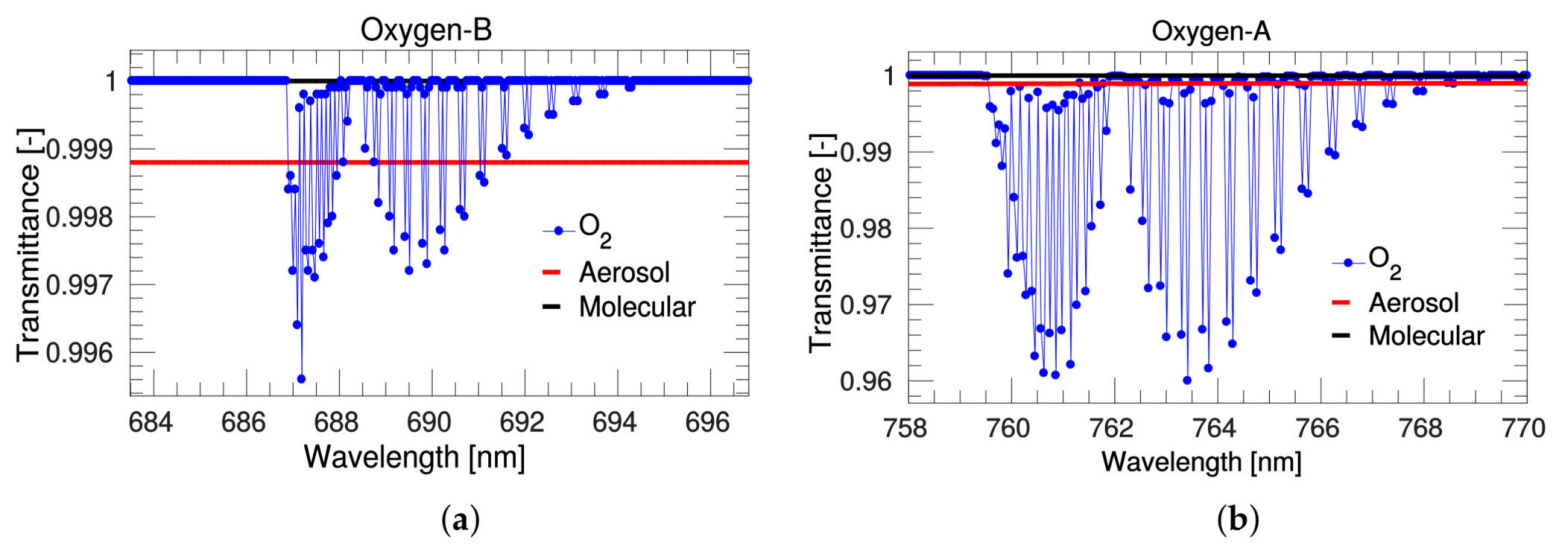
Simulated upward atmospheric transmittance from a 0 m surface elevation to a tower sensor at 10 m height, with nadir viewing geometry and sea–level pressure conditions. Transmittance has been simulated at 1 cm^−1^ spectral resolution for the O_2_–B (**a**), i.e., ~0.05 nm, and the O_2_–A (**b**), i.e., ~0.06 nm, absorption regions. Note the different Y–axis ranges used to emphasize details in both O_2_–B and O_2_–A panels.

In case of short target–sensor distances, 10 m, it is possible to: (a) neglect the contribution of the atmospheric path radiance, *L*_0_, [40]; (b) consider that the total upward atmospheric transmittance is dominated by the direct transmittance component $\left(t_{dir}^{↑}∼5t_{dif}^{↑}\right)$; and (c) assume that the contribution of the spherical albedo at TOC and at sensor level is equivalent. Therefore, the relationship between the at–sensor radiance, L, and the up–welling canopy radiance from the surface, $\bar{L}$, can be simplified as:

(3) $$L≃\left(\frac{\bar{E}}{\pi }\rho +F\right)\cdot t_{dir}^{↑}=\bar{L}\cdot t_{dir}^{↑}$$

where the *t↟_ir_* is the direct upward transmittance from the surface to the sensor level for a given viewing geometry. For the O_2_–A absorption band, the direct upward transmittance is mostly dominated by O_2_ concentration (see Figure 2). Hence, for the O_2_–A absorption band and a near target–sensor distance, it is possible to assume that $t_{dir}^{↑}∼t_{O_{2}}^{↑},t_{O_{2}}^{↑}$ being the oxygen transmittance.

In the particular case of multi–angular acquisition systems, e.g., the FUSION tower–mounted system [43], O_2_ transmittance correction becomes even more critical if the objective aims to integrate measurements over the footprint or to compare contributions from multiple acquisition geometries, since atmospheric corrections should account for the varying optical path with viewing angularity.

### 2.3 The Atmospheric Inversion Problem at High Spectral Resolution

Let us consider Equation (3), but now taking into account that radiance acquired by any sensor is provided according to its ISRF, i.e., 〈*L*〉 Therefore, radiance at TOC, $\langle \bar{L}\rangle$ could be estimated by computing:

(4) $$\langle \bar{L}\rangle =\frac{\langle L\rangle }{\langle t_{dir}^{↑}\rangle }$$

**Figure 3:**
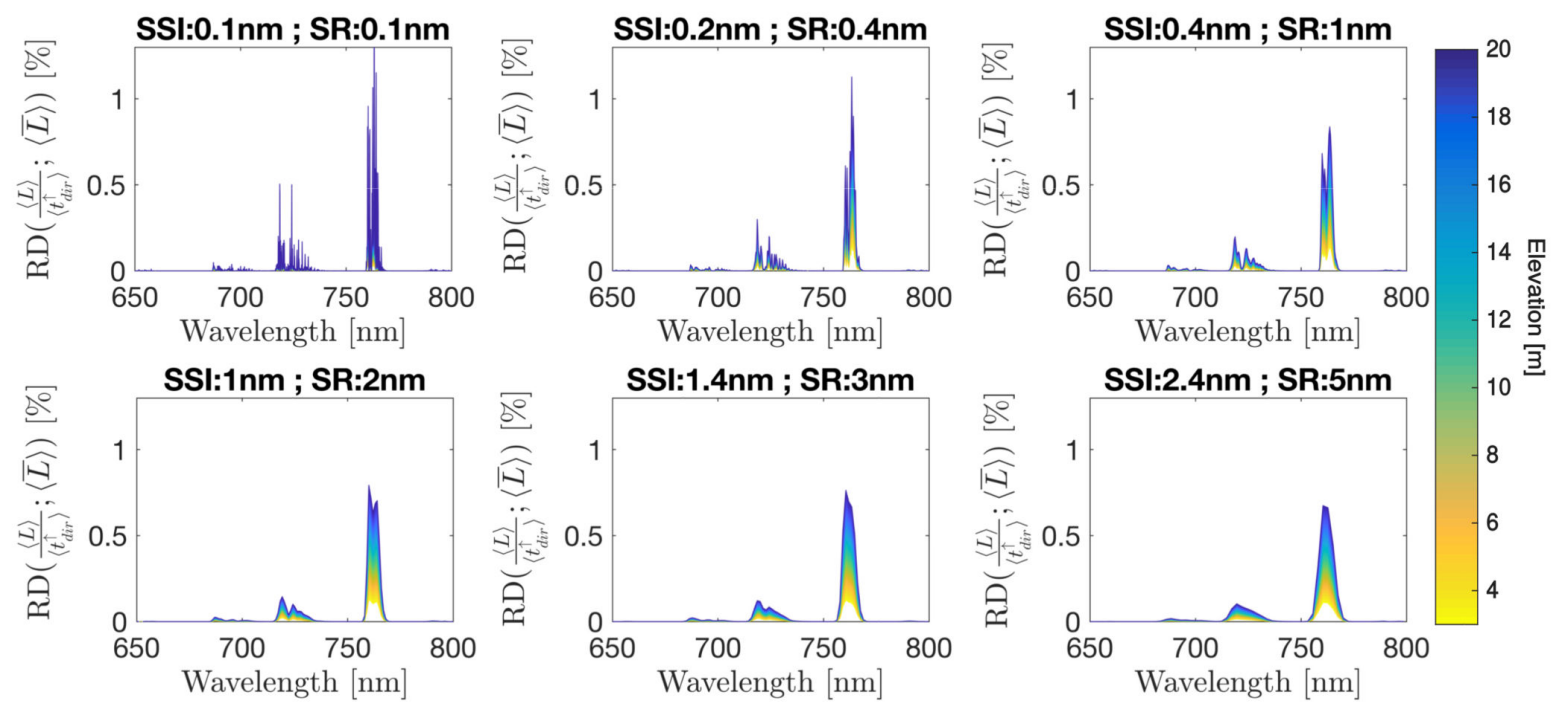
Relative difference in per cent derived from Equation (4), i.e., *RD*(%) = 100. $\left(\frac{\langle L\rangle }{\langle t_{dir}^{↑}\rangle }-\langle \bar{L}\rangle \right)/\langle \bar{L}\rangle$, covering from 3 to 20 m target–sensor distances for a range of Spectral Resolution (SR) and a Spectral Sampling Interval (SSI), as indicated in each panel.

However, Equation (4) implies convolving the atmospheric transmittance and the at–sensor radiance terms individually. This mathematical inconsistency, i.e., $\frac{\langle L\rangle }{\langle t_{dir}^{↑}\rangle }\neq \langle \frac{L}{t_{dir}^{↑}}\rangle$, is quantified in Figure 3 for a range of target–sensor distances. Notice that higher relative differences occur at the longer target–sensor distance (blue regions), because of the deeper oxygen absorption. Similarly, higher relative differences are also found with higher spectral resolution, because the narrower bands access deeper absorption lines.

Consequently, when compensating for the oxygen transmittance effects in the FLD and SFM family of retrieval strategies this fact must be taken into account, since a simple compensation of the oxygen transmittance will also introduce an error driven by the instrument spectral resolution (see Section 4).

## 3 SIF Retrieval Methods

### 3.1 FLD and SFM Methods

The family of FLD–methods, typically applied to the O_2_–A band or to Fraunhofer lines to retrieve SIF, are based on the exploitation of two or more channels inside and outside the selected absorption band. Assuming a Lambertian behaviour of surface reflectance, radiance measured from a vegetated target at surface level $\bar{L}$ can be expressed as two complementing contributions: (1) the reflected solar irradiance, and (2) the SIF emission. Mathematically, this can be expressed at a given wavelength *(λ)*:

(5) $$\bar{L}(\lambda )=\rho (\lambda )\cdot \frac{\bar{E}(\lambda )}{\pi }+F(\lambda )$$

where *Ē* is the total solar (direct and diffuse) irradiance reaching the surface, *ρ* is the Lambertian surface reflectance, and *F* is the fluorescence emission. Commonly to all FLD-family techniques, the radiances inside $\left({\bar{L}}_{in}\right)$ and outside $\left({\bar{L}}_{out}\right)$ the absorption band are related by assuming a simplified behaviour of the *ρ* and *F*. For the FLD, a constant wavelength response is assumed [19], whereas a spectral dependency is introduced for the iFLD method [22]. In view of these assumptions, the FLD and 3FLD methods can be considered as particular cases of the iFLD formulation [22]:

(6) $$F=\frac{\alpha_{R}\cdot {\bar{E}}_{out}\cdot {\bar{L}}_{in}-{\bar{E}}_{in}\cdot {\bar{L}}_{out}}{\alpha_{R}\cdot {\bar{E}}_{out}-\alpha_{F}\cdot {\bar{E}}_{in}}$$

where $\alpha_{R}=\frac{\rho_{\text{out }}}{\rho_{in}}\text{ and }\alpha_{F}=\frac{F_{\text{out }}}{F_{in}}$ are the coefficients that introduce the spectral variation of *ρ* and *F* in the absorption band region. Note that the FLD method is the particular case where *α_R_* = 1 and *α_F_* = 1 [22], and the 3FLD formulation is equivalent to the original FLD formulation when *L_out_* and *E_out_* are obtained by linear interpolation of two bands at each side of the absorption panel [21].

Unlike the simpler FLD methods, the SFM approach exploits all the spectral information around the selected absorption band [23,24], or even the complete spectral SIF range from 650 nm to 800 nm [25]. According to the selected spectral interval, reflectance and fluorescence spectra can be described by appropriate mathematical functions. Therefore, a spectral difference, *ϵ*(*λ*), exists between the modelled and observed radiance at TOC:

(7) $$\arg_{\vec{x_{\rho }},\vec{x_{F}}}\text{min}\left(\bar{L}(\lambda )-\underset{L_{MOD}}{\underset{︸}{\left(\rho_{MOD}(\lambda )\cdot \frac{\bar{E}(\lambda )}{\pi }+F_{MOD}(\lambda )\right)}}\right)$$

where $\bar{L}(\lambda )$ is the observed radiance at TOC from Equation (5), and $\vec{x_{\rho }},\vec{x_{F}}$ are vectors containing the parameters used to reproduce *ρ_MOD_* and *F_MOD_* spectral functions. For narrow spectral intervals covering the O_2_–A region, the SFM typically models *ρ_MOD_* and *F_MOD_* by polynomial (quadratic or cubic) functions [44]. Thus, the inversion process disentangles *F_MOD_* from *ρ_MOD_* by finding the coefficients of the parametric functions for *ρ_MOD_* and *F_MOD_* that minimizes *ϵ(λ)* [24].

### 3.2 O_2_ Transmittance Compensation on FLD and SFM

Following the initial FLD assumptions, i.e., *F*(λ*_out_*) ~ *F*(λ*_in_*) and *ρ*(λ*_out_*) ~ *ρ*(λ*_in_*); and introducing Equations (1) and (3) into Equation (6), SIF can be formulated as:

(8) $$F=\frac{\frac{L_{in}}{t_{in}^{↑}}\cdot \alpha_{R}\cdot E_{\text{out }}\cdot t_{\text{out }}^{↓}-\frac{L_{\text{out }}}{t_{\text{out }}^{↑}}\cdot E_{in}\cdot t_{\text{in }}^{↓}}{E_{\text{out }}\cdot \alpha_{R}\cdot t_{\text{out }}^{↓}-E_{in}\cdot \alpha_{F}\cdot t_{in}^{↓}}$$

where Equation (8) represents the improved formulation of the iFLD method to compensate for the O_2_ transmittance. Note that Equation (8) is also valid for the FLD and 3FLD methods when the spectral fluorescence and reflectance correction factors are *α_F_* =1 and *α_R_* =1, respectively. Outside the O_2_–A absorption band, upward and downward transmittances (*t*^↑^ and *t*^↓^) can be set to unity, which simplifies Equation (8). In the case where solar irradiance is measured at TOC level, then *E* · *t*^↓^ is replaced by *Ē*; and thus, no O_2_ correction needs to be applied to the solar irradiance term. Inside the O_2_–A absorption band, upward and downward oxygen transmittance can be simulated using an atmospheric radiative transfer code, such as the MODerate resolution atmospheric TRANsmission (MODTRAN, [42]), or using the HIgh–resolution TRANsmission molecular absorption database (HITRAN, [45]). Alternatively, empirical O_2_ transmittance approximations [37] as shown in Equations (9) and (10) can also be used for moderate spectral resolution (~0.22 nm) measurements:

(9) $$t(\lambda )=\text{exp}\left[-{\left(10^{C^{\prime }(\lambda )}{\left(\frac{p}{p_{0}}\right)}^{n}{\left(\frac{T_{0}}{T}\right)}^{m}U\right)}^{a}\right]$$

(10) $$U=0.7732\cdot 10^{-4}M\rho_{a}Z$$

where *p*(atm), *T*(K), *M*(ppmv) and *ρ_a_*(g·m^—3^) are the pressure, temperature, absorber concentration, air density conditions, respectively, for a particular path length height (e.g., the distance between the TOC and the sensor); and U(atm · cm) is the total absorber amount in the path length, Z(km). Finally, the subscript T_0_, p_0_ indicates the standard conditions, i.e., 273.16 K and 1 atm, and the values of the parameters *a*, *m* and *n* are set to 0.5641,0.9353 and 0.1936, respectively. See also Pierluisi et al., 1986 [37] for more details and default values typically assumed for the *C′* spectral coefficients.

When using the SFM for proximal–sensing retrievals, then the observed radiance at sensor level (L from Equation (7)), is affected by atmospheric transmittance between the TOC target and the sensor. Therefore, assuming that the solar irradiance (E) and the up–welling radiance (L), are both measured away from the surface and using the relationship from Equation (3), the modified formulation of the SFM for a first order O_2_ compensation would be:

(11) $$\arg_{\vec{x_{\rho }},\vec{x_{F}}}\text{min}\underset{\text{Surface }}{\underset{︸}{\left(\frac{L}{t_{vza}^{↑}}-\left(\frac{E\cdot t_{\text{sza}}^{↓}}{\pi }\cdot \rho_{MOD}+F_{MOD}\right)\right)}}\Leftrightarrow \arg_{\vec{x_{\rho }},\vec{x_{F}}}\text{min}\underset{\text{Sensor }}{\underset{︸}{\left(L-\left(\frac{E\cdot t_{sza}^{↓}}{\pi }\cdot \rho_{MOD}+F_{MOD}\right)t_{vza}^{↑}\right)}}$$

where the $t_{sza}^{↓}$ and $t_{vza}^{↑}$ are the (wavelength–dependent) downward and upward atmospheric transmittances. Suffixes *sza* and *vza* indicate the main dependence on the solar illumination and the sensor acquisition geometry, respectively. In Equation (11), the spectral dependency of each of the functions involved is omitted for brevity. Note that Equation (11) indicates that it is mathematically equivalent to minimizing the spectral difference between the modelled and measured radiance at TOC or at sensor level, given that the O_2_ transmittance correction is included in the formulation.

The first order O_2_ compensation techniques detailed here are addressed for measurements acquired at a few meters distance from TOC surface level. These techniques cannot be extrapolated to airborne or satellite level, where the atmospheric path radiance (L_0_) and spherical albedo (S) must be included in the atmospheric correction scheme. Furthermore, whereas O_2_ absorption affecting proximal sensing is still dominated by O_2_ gas concentration (Figure 2), as the atmospheric path increases, aerosols and molecular scattering also play an important role in this spectral region and thus must be carefully compensated.

### 3.3 Adapting an Airborne Atmospheric Correction Scheme for Proximal Sensing Data

**Figure 4:**
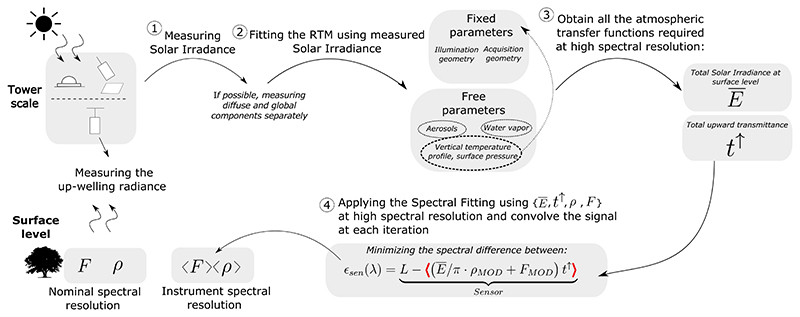
Adaptation of a standard airborne atmospheric correction scheme when applying the SFM SIF retrieval approach for proximal sensing data. Red bold angle brackets in step 4 indicate the signal to be convolved according to the ISRF.

In Section 3.2, the standard FLD and SFM formulations were modified to include a first order compensation of the O_2_ absorption effects acting on the atmospheric path between the target and the sensor. However, as pointed out in Section 2.3, the multiplication or division of spectral functions that were previously convolved according to the ISRF will result in errors, particularly in the absorption regions. In consequence, in order to compensate for the ISFR convolution effects, it is necessary to use the atmospheric functions at a finer spectral resolution than the instrument’s spectral resolution. Since this is hardly the case in practice, here we describe the adaptation of a typical airborne atmospheric correction strategy to simultaneously deal with: (1) the O_2_ absorption effects; and (2) the particular instrument ISRF, when measuring both solar irradiance and the up–welling canopy radiance for proximal sensing (Figure 4).

In essence, the idea is based on using the solar irradiance signal acquired at the same tower–mounted height (e.g., top of the tower), either using a reference panel or with an upward looking cosine corrected sensor, to fit an atmospheric Radiative Transfer Model (RTM) (steps ①) and ② from Figure 4). This way, the atmospheric state is fully characterized and the RTM can provide all the atmospheric functions required to apply the SFM with the O_2_ compensation, i.e., *ρ* and *t^d^* according to Equation (12) (step ③ from Figure 4). Note that the use of a finer spectral resolution sensor for acquiring the solar irradiance at TOC, E, will not entirely solve the problem since the upward transmittance, *t*^↑^, is still required at a high spectral resolution. Once the atmospheric functions *Ē* and *t*^↑^ are characterized, the SFM can be consistently applied at sensor level by convolving $\langle (\bar{E}/\pi \cdot \rho_{MOD}+F_{MOD})t^{↑}\rangle$ at each iteration of the minimization process in which $\vec{x_{\rho }}$ and $\vec{x_{F}}$ change (step ④) (see Equation (12)).

(12) $$\arg_{\vec{x_{\rho }},\vec{x_{F}}}\text{min}\underset{\text{Sensor }}{\underset{︸}{\left(\langle L\rangle -\langle \left(\bar{E}/\pi \cdot \rho_{MOD}+F_{MOD}\right)t^{↑}\rangle \right)}}$$

## 4 Impact of Oxygen Transmittance Compensation on Different SIF Retrieval Strategies

In order to quantify the expected improvement achieved by each of the proposed compensation techniques in Section 3, we now present a collection of tests that were developed using a set of simulated radiance spectra at varying sensor levels (from 3 m to 20 m above TOC) following Equation (3). In the radiance simulation process at sensor level, total atmospheric upward transmittance (including aerosols) and at–surface solar irradiance were simulated using the atmospheric RTM MODTRAN. In order to disentangle errors derived for SIF due to the O_2_ effects from those due to the retrieval technique, four tests were performed: (1) a high spectral resolution test assuming the knowledge of surface reflectance (Section 4.1); (2) a test using the O_2_ compensated 3FLD formulation (Section 4.2); (3) a test using the O_2_ compensated SFM formulation (Section 4.3); and (4) a final test adapting an airborne atmospheric correction strategy by coupling the O_2_ transmittance with the compensation of the ISRF convolution on the SFM (Section 4.4).

### 4.1 High Spectral Resolution

**Figure 5:**
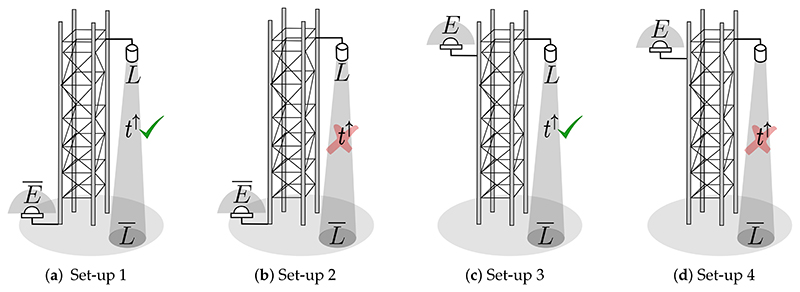
Diagrams in (**a**–**d**) corresponding to the instrument set–up configurations (1), (2), (3) and (4), respectively, described in Section 4.1. (**d**) represents the common configuration of tower–mounted instruments to measure SIF at tower–scale. In some cases, downward looking sensors mounted at the tower’s top allow multi–angular data collections. Down–welling solar irradiance is typically acquired with upward looking hemispherical or conical systems or by measuring a reference panel with a downward looking sensor. Here *t*^↑^ is the upward atmospheric transmittance.

An initial simulated experiment was conducted to estimate the impact of ignoring oxygen effects on the retrieved SIF at a high spectral resolution of 0.06 nm, regardless of the retrieval technique implemented. To do so, four instrumental configuration set–ups were evaluated: (1) *L* corrected for target–sensor O_2_ absorption and *Ē* measured at TOC (Figure 5a); (2) *L* **not** corrected for target–sensor O_2_ absorption and *Ē* measured at TOC (Figure 5b); (3) *L* corrected for target–sensor O_2_ absorption and *E* measured at sensor level on a tower (Figure 5c); and (4) *L* **not** corrected for target–sensor O_2_ absorption and *E* measured at sensor level on a tower (Figure 5d).

In all cases, SIF was estimated by isolating *F* from Equation (3), assuming the surface reflectance spectrum was known. Thus, the resulting expression used to estimate SIF in each experimental set–up was:

- Set–up (1): $F=L/t_{O_{2}}^{↑}-(\bar{E}\cdot \rho )/\pi$
- Set–up (2): *F* = *L* — ($\bar{E}$ · *ρ*)/*π*
- Set–up (3): $F=L/t_{O_{2}}^{↑}-(E\cdot \rho )/\pi$
- Set–up (4) : *F* = *L* — (*E* · *ρ*)/*π*

**Figure 6:**
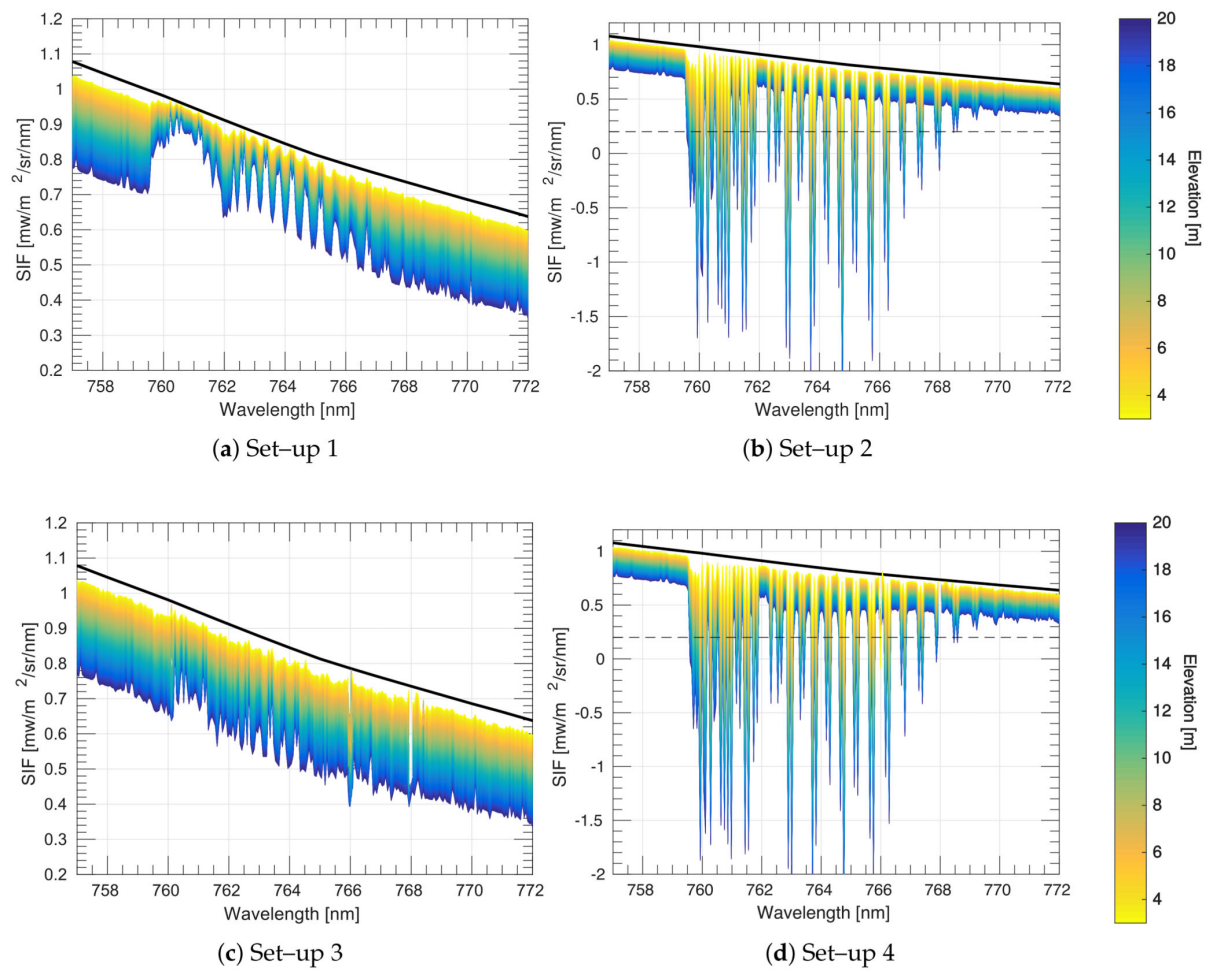
Retrieved SIF in the O_2_–A region under conditions configured for instrumental set–ups (1–4). Figure (**a**) Upward O_2_ transmittance corrected ($t_{{\text{O}}_{2}}^{↑}$) and solar irradiance measured at surface level (E); (**b**) No upward O_2_ transmittance corrected and solar irradiance measured at surface level (E); (**c**) Upward oxygen transmittance corrected ($t_{{\text{O}}_{2}}^{↑}$) and solar irradiance measured at sensor level *(E);* (**d**) No upward O_2_ transmittance corrected and solar irradiance measured at sensor level (E). The black dashed line in (**b**), and (**d**) shows the lower y–axis limit set in (**a**), and (**c**) Notice that the SIF ranges vary for the panels, especially for (**a**,**c**) versus (**b**,**d**).

The retrieved O_2_–A band SIF from all set–up configurations (1 – 4) was obtained for a nadir observation geometry and covering sensor mounted–heights from 3 m to 20 m on a tower (Figure 6). At greater sensor heights above the TOC, the impact of aerosol and molecular oxygen on retrieved SIF becomes more critical.

**Figure 7:**
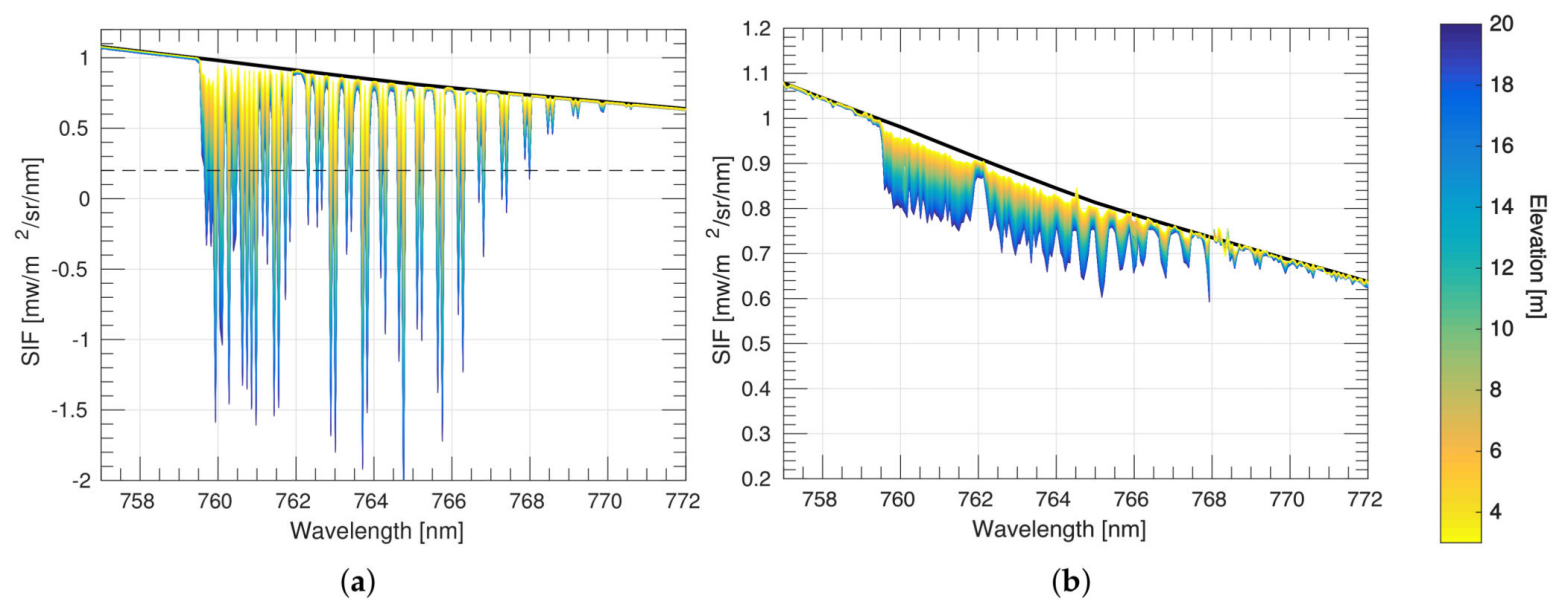
SIF in the O_2_–A region was estimated by: (**a**) correcting the at–sensor radiance for the aerosol transmittance ($t_{\text{aer }}^{↑}$) and measuring the solar irradiance at TOC surface level; (**b**) correcting the at–sensor radiance for the total transmittance (t↟) and measuring the solar irradiance at sensor level. The black dashed line in (**a**) shows the lower y–axis limit established in (**b**). Notice that (**a**,**b**) have different SIF ranges.

According to Figure 6, estimated (coloured lines) and reference (black solid line) SIF values inside the O_2_–A absorption band are reasonably close, especially for bands deep inside the absorption dip, and particularly in cases where the O_2_ correction was applied, i.e., set–up configurations (1) and (3). However, when no O_2_ absorption correction on the target–to–sensor optical path is applied, i.e., set–up configurations (2) and (4), then retrieved SIF is highly underestimated. Outside the O_2_–A absorption region, retrieved SIF is biased compared with the reference SIF (black solid line) for all the scenarios, and this effect increases as the sensor height increases. This is attributed to limiting the correction of the atmospheric transmittance (*t*^↑^) to only the O_2_ transmittance ($t_{{\text{O}}_{2}}^{↑}$). This effect is demonstrated in Figure 7a where SIF was estimated by correcting the at–sensor radiance for only the aerosol transmittance ($t_{\text{aer }}^{↑}$). As it can be observed, the earlier existing bias in the spectral region outside of the O_2_ absorption band has disappeared. In addition, in Figure 7b, we evaluated the impact of using the solar irradiance at sensor level instead of at TOC. To do so, SIF was estimated by correcting the at–sensor radiance using the total atmospheric transmittance (*t*^↑^). This case is similar to the set–up configuration 3, but residuals in the estimated SIF are now exclusively caused by the at–sensor measured solar irradiance. In both cases of Figure 7, because the at–sensor radiance has been corrected for the total or aerosol transmittance, there are no residuals caused by aerosols out of the absorption band (i.e., no bias is observed).

Likewise, an analogous analysis was conducted for the O_2_–B region and for a wider spectral range from 650 nm to 800 nm. Corresponding figures are presented as part of the supplementary material of this paper (Figures S1 and S2 in Supplementary Materials). Additionally, information regarding the input parameters used in the MODTRAN RTM as part of the simulation, as well as the surface reflectance and fluorescence spectra used are detailed in Appendix A.

### 4.2 Oxygen Compensated 3FLD

**Figure 8:**
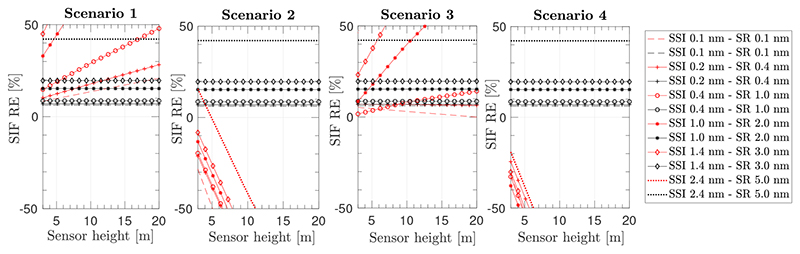
Relative error (%) on retrieved SIF in the O_2_–A region when applying the 3FLD method according to Equation (8) for a range of spectral resolutions, applied to set–up configurations 1–4 (red symbols and lines) from left to right panels, respectively. The SIF relative error obtained when applying the 3FLD at TOC surface level is indicated with black symbols and lines.

A second simulation experiment was performed by varying the sensor height above TOC (from 3 m to 20 m), and the instrument spectral configuration for the 4 set–up configurations as described in Section 4.1. In this section, we applied the 3FLD method with the approximation to compensate for the O_2_–A transmittance effect, as given in Equation (8). The selected sensor specifications, i.e., Spectral Resolution (SR) and the Spectral Sampling Interval (SSI), are listed in the legend of Figure 8 and are based on configurations evaluated by [46].

Figure 8 shows the performance of the 3FLD method under set–up configurations 1–4 for different sensor specifications. Since the 3FLD method depends strongly on the wavelength locations, especially for the wavelength at the bottom of the O_2_–A absorption region, we have kept the central wavelength of the band located at the bottom of the O_2_–A absorption region constant at 760.6 nm in all of the sensor specifications evaluated. Consistent with results shown in Figure 6, as the sensor height above TOC increases, set–up configurations 2 and 4 underestimate the retrieved SIF and produce negative values. For set–up(s) 1 and 3, where O_2_ transmittance between the target and the sensor is corrected, SIF was overestimated as the sensor height increases and as the sensor resolution decreases. In addition, according to Figure 6, we could expect that SIF estimations from set–up 1 were more accurate (provide lower relative errors [%]) than from set–up 3. However, this is not the case. This can be attributed to a compensation between the overestimation produced by the 3FLD technique and the stronger SIF underestimation produced in set–up 3 by measuring the solar irradiance at sensor level. Note, that for all the configurations, SIF was also retrieved at TOC level using the 3FLD method to identify the relative error baseline for each of the sensor configurations evaluated (black symbols and lines in Figure 8), for comparison.

SIF relative errors in Figure 8 have been limited to a range of ±50%. For set–up 4, where no O_2_ compensation is applied, the application of the 3FLD method produces high underestimations of the SIF values, even for sensors located as close to the surface as 3 m, regardless of the instrument configuration used. For configurations 1 and 3, only in the particular cases where the instrument acquires the signal at high spectral resolution (i.e., SSI < 0.2 nm and SR < 0.4 nm), does the compensation for the O_2_ effects lead to SIF relative errors less than 30% for all sensor heights.

### 4.3 Oxygen Compensated SFM

This section assesses the performance of the O_2_ compensated SFM for a range of sensor heights above TOC (from 3 m to 20 m) on the O_2_–A absorption region and for the same instrument configurations evaluated in Section 4.2. For a specific sensor, the SFM depends strongly on: (1) the considered wavelength interval; (2) the use of weighting functions; and (3) the mathematical functions used to model the fluorescence, *F*, and the reflectance, *ρ*, spectra [24].

**Figure 9:**
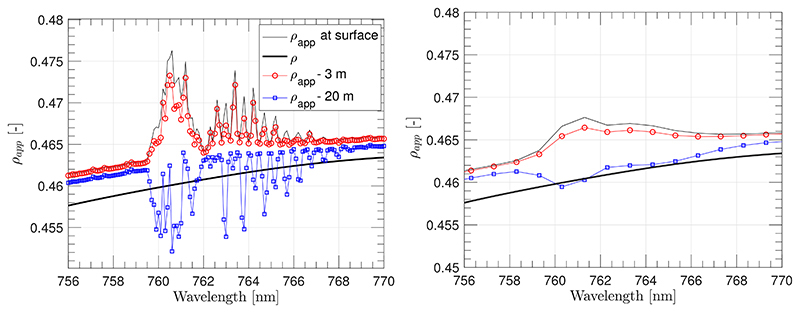
Apparent reflectance (*ρ_app_*) derived at: (**a**) high (SSI 0.1 nm - SR 0.1 nm); and (**b**) low (SSI 1 nm–SR 2 nm) instrument resolutions for 3 m (red circles) and 20 m (blue squares) sensor heights above the TOC. The solid thin black lines correspond to the *ρ_app_* derived at surface level. The solid thick black line corresponds to the surface reflectance used here as a reference.

According to the results obtained in Meroni et al., 2010 [24] for noise–free data, the best SFM performance was obtained by modelling SIF (F) as a quadratic function and reflectance (*ρ*) as a cubic function for a narrow spectral interval ranging between 759.3–762.0 nm, without any weighting function. Due to multiple spectral configurations considered here, in order to ensure that enough bands are selected (specially for the lower resolutions evaluated), we have expanded the selected spectral interval to 759.3–767.5 nm (which corresponds to the third range interval described in [24]). For the SFM calculation, the first guess of the *F* polynomial coefficients was estimated by fitting the reference SIF spectrum to a quadratic function and distorting the derived coefficients by 10% of their value. Conversely, the first guess of the *ρ* polynomial coefficients was estimated by fitting the apparent reflectance spectrum *ρ_app_* to a cubic function, in the spectral region around the O_2_–A, but avoiding the absorption band. The apparent reflectance spectrum, *ρ_app_*, was obtained as the ratio between the upward radiance and the incoming solar irradiance measured at sensor level. In Figure 9, *ρ_app_* at the highest and lowest spectral resolution that we evaluated are presented for “at–TOC” surface level, and at 3 m and at 20 m heights. It can be observed that with increasing sensor height, peaks in the apparent reflectance become lower. In the particular case of the 20 m sensor height, peaks in the *ρ_app_* have been transformed into depressions.

**Figure 10:**
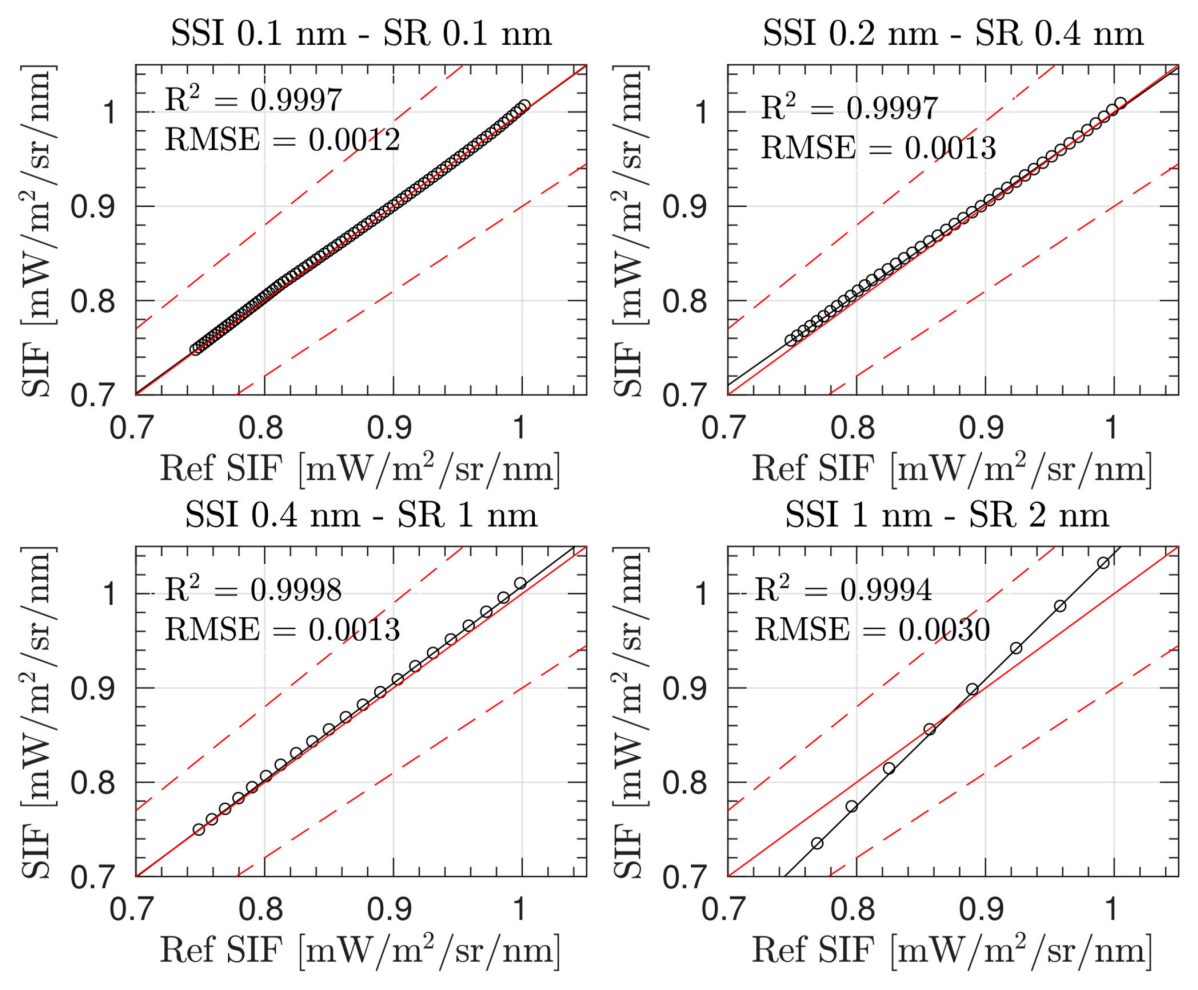
Scatter plots of the reference spectra and estimated SIF spectra values (black circles) in the interval between 759.0–767.5 nm, for different instrumental spectral resolutions (SR) and sampling interval (SSI). Estimated SIF was derived by applying the SFM at TOC surface level. Red solid line represents the 1:1 line, and red dashed lines define the region for a SIF relative error lower than 10%.

Prior to showing the impact of the O_2_ effects on the modified SFM formulation, we present as a reference point (Figure 10), the level of accuracy of the retrieved SIF when the SFM is applied at TOC surface level for the given surface reflectance and SIF spectra, with the polynomial functions used to model these signals, and the selected spectral interval. As can be seen in Figure 10, relative errors are lower than 10% for high resolution spectrometers. This error increases with decreasing spectral resolution, i.e., for sensors with wider bands.

**Figure 11:**
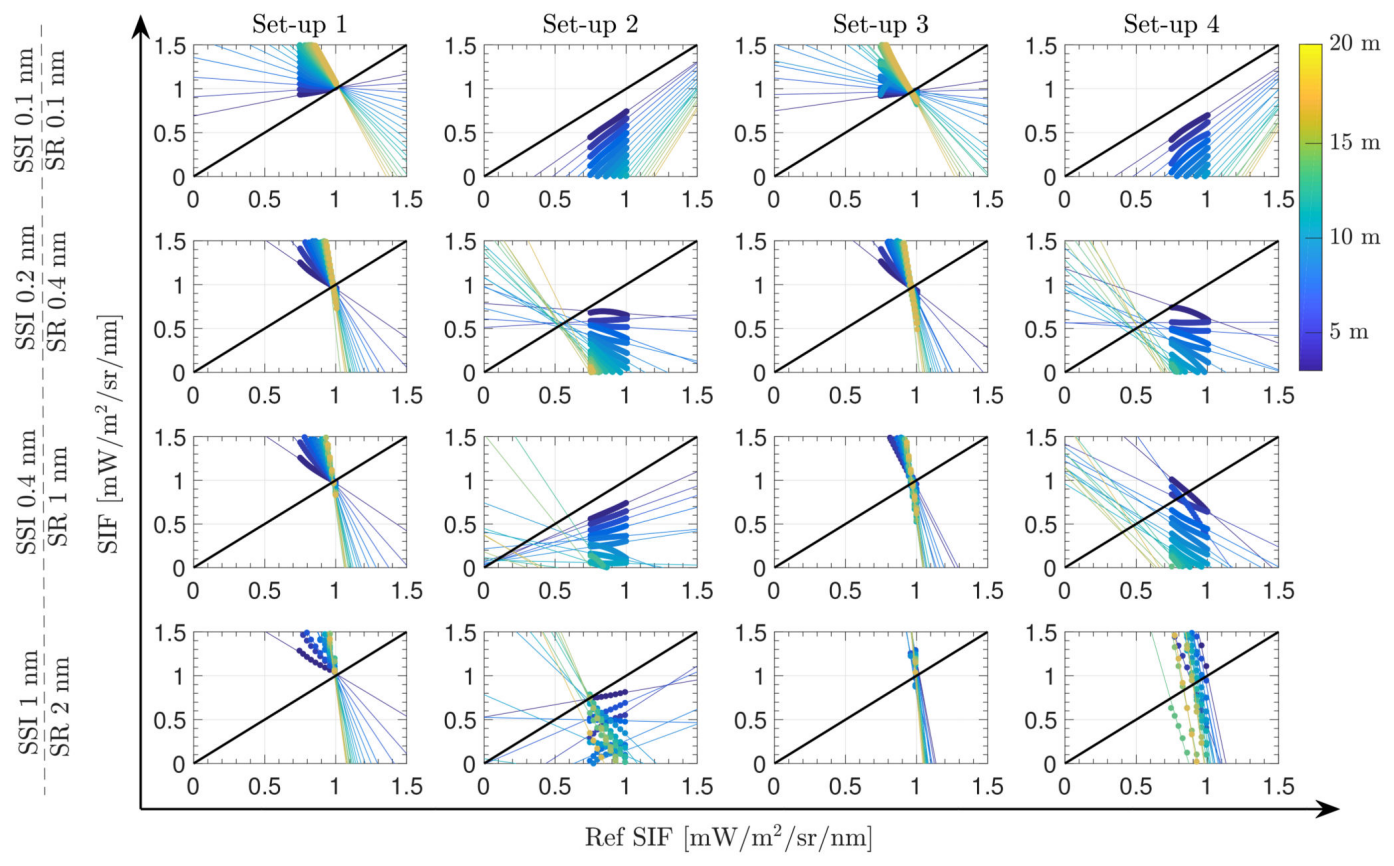
Scatter plots of reference SIF versus estimated SIF spectra in the interval between 759.0–767.5 nm using the SFM retrieval approach and O_2_ compensation while ignoring instrument spectral convolution effects for set–up configurations 1–4. Simulations are shown for different instrument resolutions (rows 1–4) and sensor heights between 3 m to 20 m (colour scale). Solid lines are regression lines. Actual estimations are presented as circles.

Therefore, following the same configuration (i.e., same spectral interval and polynomial functions to model *ρ* and *F*), the performance of the SFM is presented for the case when O_2_ transmittance effects are compensated using Equation (11) (Figure 11).

Figure 11 shows the estimated SIF for each of the above–evaluated set–ups 1–4. Errors on retrieved SIF are derived due to: (1) modelling the *ρ* and *F* signals as a cubic and a quadratic function, respectively; (2) introducing the O_2_ transmittance compensation but ignoring the effects of multiplying atmospheric functions with absorption features already convolved (mentioned in Section 3.3); and (3) not restricting the modelled functions to realistic values. In set–ups 1 and 3, where the upward O_2_ transmittance from target to sensor is corrected ($t_{O_{2}}^{↑}$) the estimations of values close to 0.97 [mW/m^2^/sr/nm] (which corresponds to the SIF reference value at the bottom of the O_2_–A band, see Figure A1) are accurately retrieved. However, estimated SIF values far away from the deepest region of the oxygen band are not successfully derived (not following the 1:1 line) due to not properly considering the convolution by the ISRF in the formulation. In the cases of set–ups 2 and 4, neither the values at the bottom of the O_2_–A band nor nearby vicinities have been successfully retrieved.

### 4.4 Airborne Atmospheric Correction Scheme Applied to Proximal Sensing Data: O_2_ and ISRF Compensated SFM

As it has been demonstrated during this section, O_2_ transmittance effects must be compensated when retrieving SIF from proximal remote sensing data. However, significant errors in estimating SIF can occur, even when applying an O_2_ transmittance compensation factor (or spectrum) in the formulations of the 3FLD (or SFM) approach(es). Thus, here we assess errors derived in the estimated SIF under scenario 1 when following the strategy detailed in Figure 4, which properly includes the ISRF convolution as part of the SFM minimization process.

**Figure 12:**
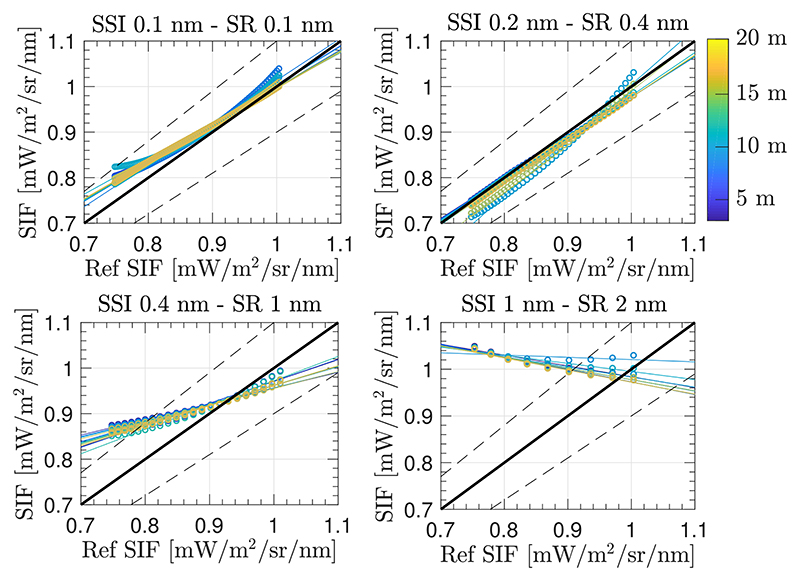
Scatter plots showing reference SIF versus estimated SIF in the interval between 759.0–767.5 nm, using the proposed SFM retrieval approach to compensate for oxygen effects while also accounting for the instrument spectral convolution effects, for different instrument spectral resolutions and sensor heights above TOC between 3 m to 20 m (colour scale). Coloured solid lines are regression lines for the range of sensor heights evaluated while actual estimations are marked as circles. The black solid lines represent the 1:1 line and dashed lines define the areas with a SIF relative error lower than 10%.

Figure 12 shows the estimated SIF at multiple sensor heights above the TOC when applying the proposed strategy (adaptation from classic airborne correction strategies) and considering the same conditions presented in Section 4.3, i.e., the same spectral interval and the same polynomial functions used to model *F* and *ρ*. Although higher relative errors are derived at lower spectral resolution, errors have significantly improved in comparison to the situation described by set–up 1 from Section 4.3, where the O_2_ compensated SFM approach was used while ignoring the effect of multiplying spectrally convolved functions. Therefore, in this case a successful SIF estimation was achieved, including those values that were not close to the bottom of the O_2_–A, i.e., SIF from 0.7–1 [mW/m^2^/sr/nm], for high spectral resolution cases.

**Figure 13:**
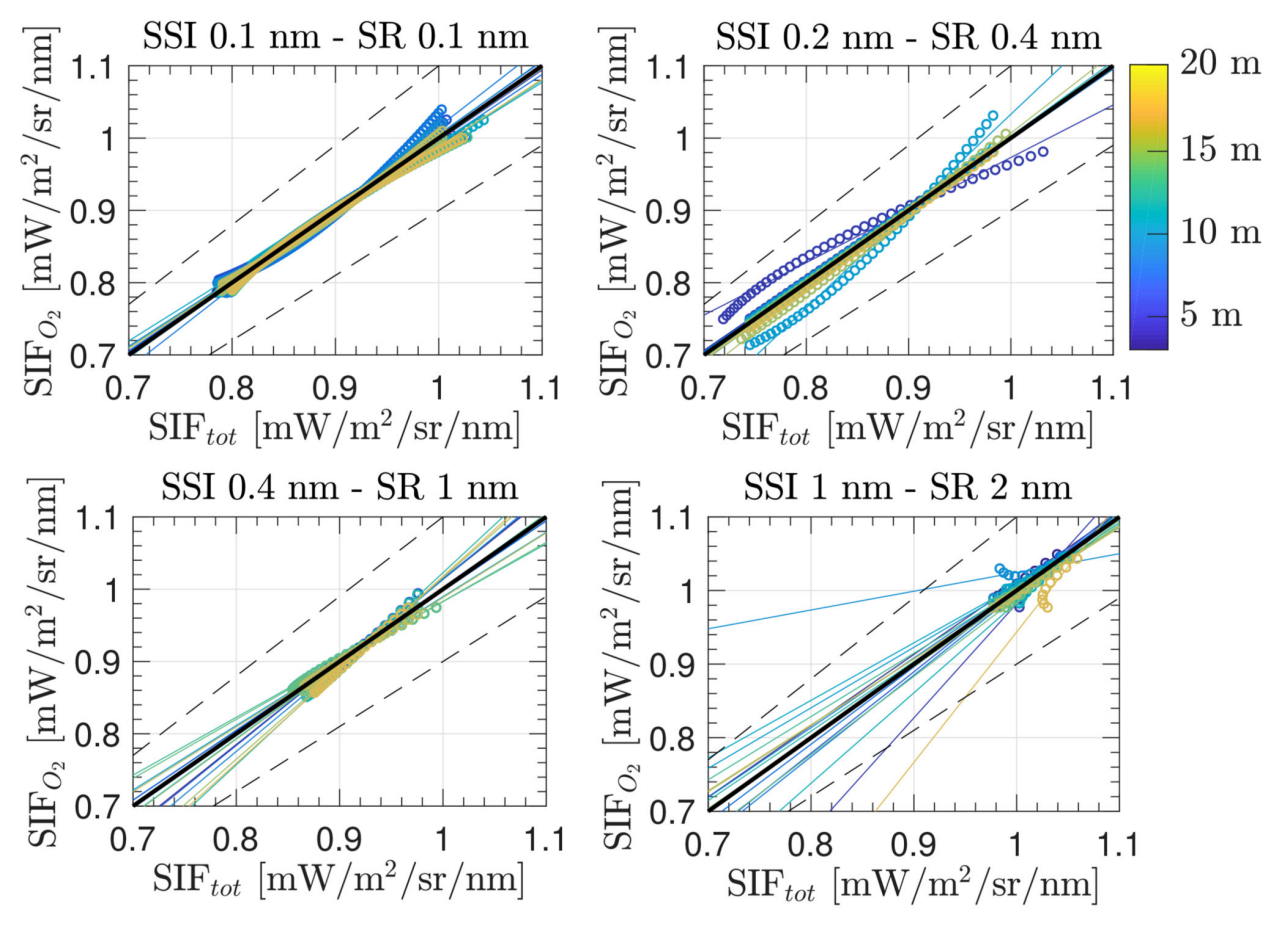
Scatter plots of estimated SIF using the proposed SFM retrieval approach to compensate (1) for oxygen effects and (2) for total atmospheric transmittance, accounting in both cases for the instrument spectral convolution effects at different instrument resolutions and sensor heights between 3 m to 20 m (colour scale). Coloured solid lines are regression lines for the range of sensor heights evaluated while actual estimations are marked as circles. The black solid lines represent the 1:1 line and dashed lines define the areas having a SIF relative error lower than 10%.

Finally, in order to evaluate the impact of excluding the aerosol compensation on the upward transmittance from the TOC level to the sensor, we quantified the impact of replacing the O_2_ transmittance with the total atmospheric transmittance function, i.e., replacing $t_{O_{2}}^{↑}$ by *t^↟^*. Since the accuracy improvement achieved for estimated SIF was not significant, to better show the slight gain, Figure 13 presents a scatter plot between estimated SIF (labelled as *SIF_O_2__*), with compensation only for O_2_ transmittance, versus compensation for the total atmospheric transmittance (labelled as *SIF_tot_*).

For more details, the Supplementary Material also presents a scatter plot relating reference SIF and *SIF_tot_* spectra (Figure S3). In addition, since the SFM also provides the reflected surface spectrum, scatter plots comparing reference and estimated surface reflectance when compensating for either oxygen transmittance only, or for total atmospheric transmittance, are also included in the Supplementary Material (Figures S4 and S5). Thus, according to this analysis, the adaptation of an airborne strategy for a few meters of target–sensor distance using the SFM approach, and only compensating for O_2_ absorption and ISRF convolution effects, would be sufficient to accurately estimate SIF on sensors with a SR < 1 nm.

## 5 Temporal Analysis on Temperature and Pressure Environmental Conditions

As previously pointed out, when using a RTM the accuracy of the retrieved SIF will strongly depend on the accuracy achieved in modelling the atmospheric conditions. As concluded in Section 4, oxygen transmittance compensation becomes essential to accurately estimate SIF for proximal sensing. In the particular case of conducting a long temporal data series analysis of measured SIF from tower, oxygen transmittance variations caused by seasonal *p* and *T* changes within the year must also be carefully considered. As an illustrative example, we analysed the expected variation, first for the oxygen transmittance and then for the acquired at–sensor radiance, caused by changes in *T* and *p* conditions (see Appendix B for details). In this example, we reproduced the radiance variations that would be observed by an instrument mounted on a tower located at the Hyytiälä Forestry Field Station in Finland, due only to changes in the meteorological conditions while keeping the surface properties, i.e., reflectance and emitted SIF, invariant. The rationale behind keeping the surface properties invariant is to evaluate changes in sensor radiance exclusively attributed to changes in the meteorological conditions. This is because we were interested in whether these pressure and thermal influences on O_2_ transmittance could be incorrectly translated into perceived changes in the retrieved SIF signal if not appropriately compensated. Thus, for the at–surface level we took the reference surface reflectance and SIF spectra shown in Figure A1. For the atmospheric simulation we did the exercise in three steps: (1) taking the *T* and *p* registered in Hyytiälä, we computed the transmittance following the empirical approximations presented in Equations (9) and (10); and (2) the resulting transmittance spectra were scaled to a high resolution MODTRAN spectra (0.1 cm^−1^). Finally, in (3) we used the MODTRAN oxygen transmittance spectra to compute sensor radiance over a full one year period using Equation (3).

Regarding the simulation protocol, while *T* was directly measured at the top of the tower (~30 m), air pressure was measured at surface level. Therefore, surface pressure at the top of the tower was computed following Equation (13), resulting from combining the hydrostatic equation together with the ideal gas law, assuming the measured total pressure as dry air pressure.

(13) $$p_{\text{sen}}=p\cdot \exp\left(\frac{gM_{0}Z}{R_{0}T}\right)$$

In Equation (13), *p_sen_* is the pressure at sensor level, *p* and *T* are the pressure and T temperature measured at TOC surface level, *M*_0_ is the molar mass of the dry air, *R*_0_ is the ideal gas constant, and *g* is the standard gravity constant. In this simulation exercise, we evaluated how variations in the meteorological conditions impacted on the O_2_ transmittance locally. Since the selected Hyytiälä tower height is around 30 m tall, about 15 above the evergreen forest canopy that it monitors, and assuming nadir acquisition geometry, we fixed the optical path as 15 m, i.e., the Z term in Equation (10).

**Figure 14:**
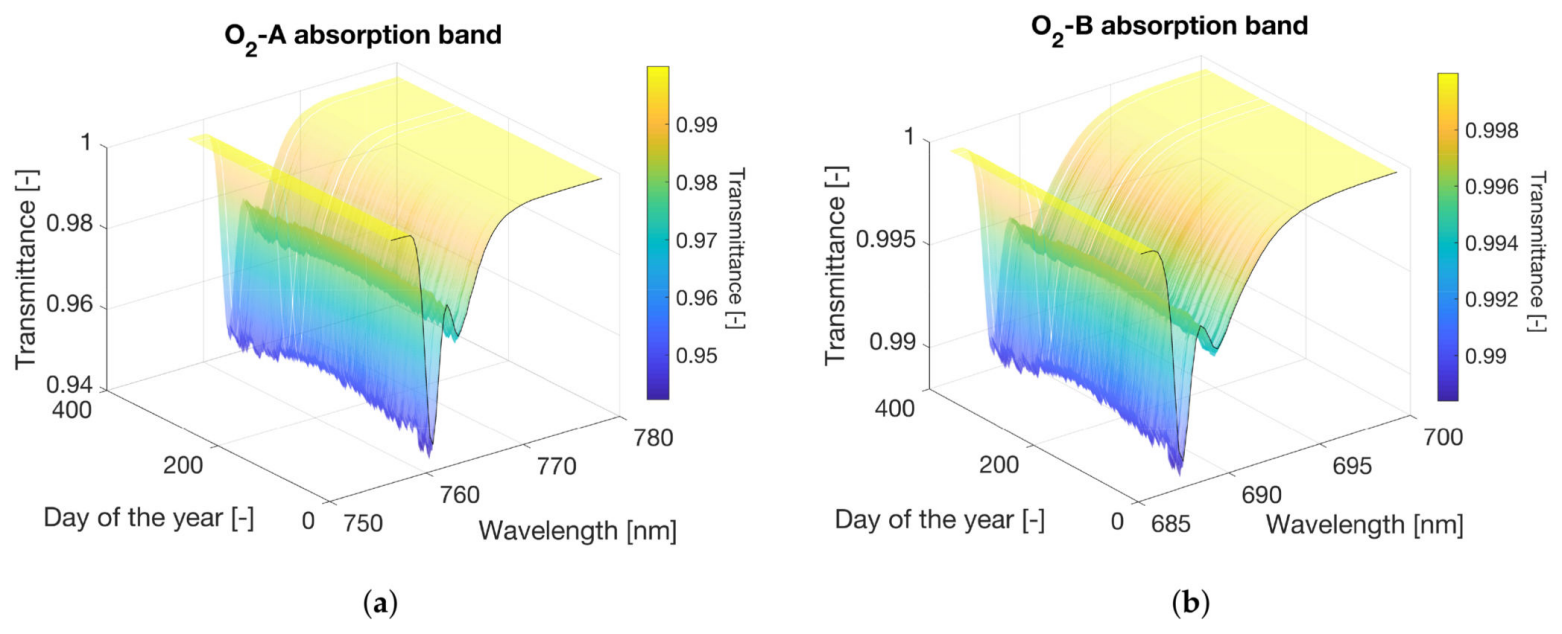
Spectral oxygen transmittance variation for the O_2_–A (**a**) and O_2_–B (**b**) absorption bands for the *T* and *p* conditions registered at 30 m tall tower at the Hyytiälä Forest Field Station in Finland.

Figure 14 shows oxygen molecular transmittance in the O_2_–A and O_2_–B absorption bands caused by *T* and *p* changes throughout a full year (2016) for the tower geometrical configuration in the Hyytiälä Forestry Field Station. As observed, the band depth in both, the O_2_–A and O_2_–B regions tracks the expected thermal dynamics related to seasonality, i.e., as *T* increases from winter minima (DOY 0, ~350) to reach maximum values in the summer season (e.g., *DOY*, ~180), the O_2_ absorption band depth becomes shorter. For the O_2_–A band, the seasonal change in transmittance is 0.01 [−] (Figure 14a), whereas for the O_2_–B case is only 0.002 [—] (Figure 14b).

**Figure 15:**
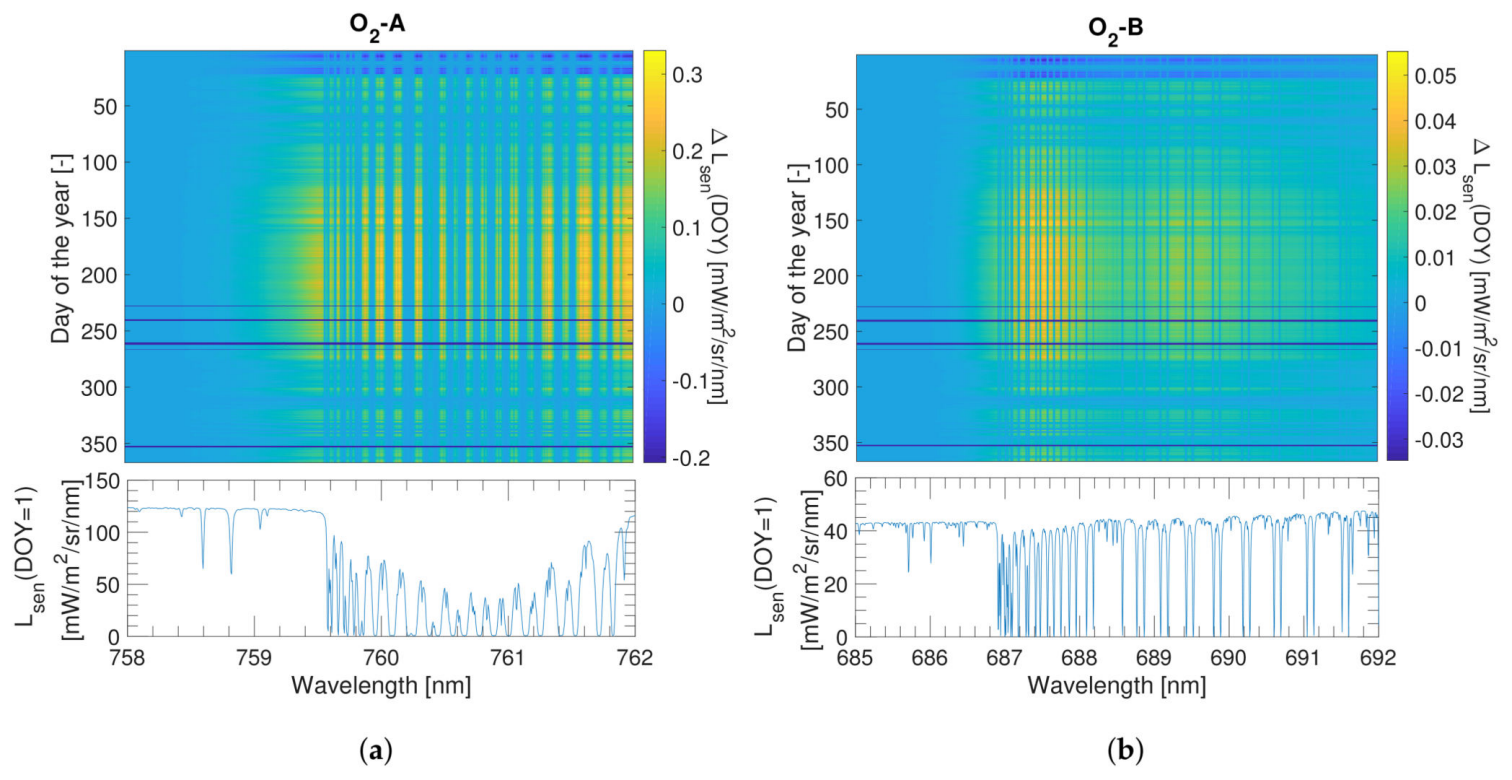
Variation in radiance units computed as *L_sen_* (DOY)-*L_sen_* (DOY=1) for the O_2_–A (**a**) and the O_2_–B (**b**) absorption regions. The acronym DOY refers to the Day Of Year from 1–365.

Now that we have shown a temperature and pressure–driven seasonal change in O_2_ transmittance at the Hyytiälä tower site during 2016, we next computed the expected at–sensor radiance variations for the O_2_–A and O_2_–B absorption regions (Figure 15) using MODTRAN simulations at the highest spectral resolution (~0.006 nm of SSI in the O_2_–A region). As observed, for the O_2_–A absorption band, seasonal changes in radiance units reached maximum values of 0.25 [mW/m^2^/sr/nm], which represents around 30% of the SIF signal at the O_2_–A band used as a reference in this example. Conversely, radiance barely reached 0.04 [mW/m^2^/sr/nm] for the O_2_–B region, representing less than 2% of the SIF signal at the O_2_–B band. This exercise indicates that SIF retrievals must include *T* and *p* measurements as part of the O_2_ compensation strategy (for the O_2_–A band) to avoid retrieved SIF over or underestimates due to environmental effects.

## 6 Discussion

### 6.1 Ground–Based Validations

Accurately measuring SIF variations due to changes in the environmental conditions can facilitate its interpretation and constrain SIF–GPP relationships. Recent studies reported a correlation between SIF and GPP by combining GPP data acquired either from Eddy–Covariance (E–C) flux towers or satellites, with satellite–derived SIF from the Greenhouse Gases Observing Satellite (GOSAT) [47,48], the Global Ozone Monitoring Mission–2 (GOME–2) [7,49–51] and with the Orbiting Carbon Observatory–2 (OCO–2) [8]. However, some concerns regarding the ecophysiological basis between SIF and GPP relationship under varying environmental conditions are still present in the scientific community [52,53], and particularly for the gap in the mechanistic understanding between SIF–GPP temporal short–term and spatial small–scale mechanisms [54]. In this respect, systematic SIF tower based measurements can play a key role: (1) to validate SIF maps derived at a global scale from satellite; (2) to analyse the impact of vegetation structure on emitted SIF; and (3) to better understand the existing relationship between SIF and other energy fluxes such as the GPP, the ecosystem respiration, latent and sensible heat fluxes, etc., which are typically related products indirectly derived from E–C flux towers.

At proximal sensing scale, the idea of linking ground–based remote sensing measurements to ecosystem CO_2_ flux data has been addressed in the past by many international initiatives, such as the SPECNET [55], and the European cost actions EUROSPEC ES0930 [5] and the OPTIMISE–ES1309 (http://optimise.dcs.aber.ac.uk/) networks. These projects have explored the use of proximal passive optical remote sensing data of ecosystems whereby carbon and water vapor fluxes are estimated at research–tower sites by E–C techniques. In particular, these network initiatives have focused on analysing, comparing and standardizing measurement protocols; while also promoting the design, testing, and development of new optical instrumentation. However, when SIF is one of the biophysical parameters under study, additional critical attention must be drawn to processing strategies as well as to instrument and measurement protocols. For instance, while instrument specifications needed to determine some biophysical remote sensing indexes such as the Normalized Difference Vegetation Index (NDVI) [56] or the Enhanced Vegetation Index (EVI) [57], can be easily reached; measuring SIF from passive remote sensing techniques is still quite challenging. In terms of the instrument specifications, high spectral resolution spectrometers with high signal to noise ratio are generally required, but these instruments are more expensive and more difficult to maintain [46]. In terms of data processing, as demonstrated in this paper, atmospheric absorption effects cannot be ignored, even when measuring data at a few meters distance from the TOC target. In this study, we demonstrated the need for compensating O_2_ molecular absorption when measuring SIF using proximal remote sensing techniques inside the O_2_–A absorption band. Since oxygen absorption is proportional to surface pressure, even a few meters distance between the TOC target and the sensor strongly impacts the retrieval of the weak fluorescence signal. Thus, it would be of great interest not only to seek a standardized measuring protocol in future experimental projects, but also to standardize common data processing strategies that deal with the compensation of O_2_ transmittance, the ISRF convolution effects, and the changing environmental conditions related to thermal and surface pressure dynamics, *T* and p. In addition, to avoid inter–calibration issues between different instruments acquiring down–welling and up–welling radiances, the use of a single spectrometer is also recommended. In line with the use of a single spectrometer, in the recent years dual fields of view instruments have been developed that guarantee quasi–simultaneous measurements of down–welling and up–welling radiation [4].

### 6.2 The Case Studies for Tower–Mounted Sensor Measurement Protocols

This demonstration began with four cases, analysing a set of simulated noise–free set-up configurations in order to distinguish between errors derived from the SIF retrieval technique and errors caused by O_2_ absorption effects: (1) using high–spectral resolution data and assuming the knowledge of surface reflectance; (2) compensating O_2_ effects using the 3FLD technique; (3) compensating O_2_ effects using the SFM strategy; and (4) adapting an airborne atmospheric correction scheme and coupling it with the SFM. According to case (1) (Section 4.1), the need to correct for oxygen absorption effects was clearly demonstrated. It was shown that correcting the acquired radiance for the upward oxygen transmittance is critical, as is acquiring solar irradiance at the tower–mounted sensor level instead of at TOC, which negatively impacts the estimated SIF. In this case, we made use of high spectral resolution data and SIF was derived by simply isolating *F* from Equation (3) according to the different experimental configuration set-up considered. In this respect, no extra ISRF convolutions were applied in this example. In case (2) (Section 4.2), the effect of compensating the 3FLD method for oxygen absorption was assessed. We demonstrated that this technique leads to high errors even in those set–up experimental configurations where the oxygen upward transmittance is corrected (set–up 1 and set–up 3). Many of those large errors were associated with negative SIF retrieval estimates, explaining the reason for this common problem in field studies. Compared to experimental results reported by Daumard et al. [10], oxygen correction in the O_2_–A region when applying the 3FLD method for a 20 m sensor height with a SR of 0.4 nm, impacts the estimated SIF around ~5% of its value. However, in our simulated study the analogous example (Figure 8, set–up 1, SR of 0.4 nm) would lead to errors in retrieved SIF of around 20% when comparing estimated SIF at sensor with SIF at TOC level. Although the significant difference reported in relative errors (from 5% to 20%), in absolute terms estimated errors in both studies are consistent, leading to a slight difference of ~0.1 mW/m^2^/sr/nm due to the contrasting SIF values at O_2_-A used in Daumard et al. (2 mW/m^2^/sr/nm) and in this study (1 mW/m^2^/sr/nm).

With regards to case (3) (Section 4.3), the compensation of the oxygen transmittance effects in the SFM delivered interesting results. As expected, more accurate SIF estimations were found in experimental set–up 1 and set–up 3, where upward oxygen transmittance is corrected. However, for all the configurations evaluated, our results indicated that estimated SIFs outside the deepest region of the O_2_–A band were biased. The observed shift in the slope of scatter plots shown in Figure 11, which affects all set-up configurations is attributed to the ISRF convolution of each of the individual terms that compose Equation (11). Thus, case (3) becomes an interesting strategy when focusing on quickly deriving a SIF value corresponding to the deepest region of the O_2_–A band, and therefore avoiding the use of any RTM. In this case, we can avoid the use of any atmospheric RTM by modelling the oxygen transmittance using the HITRAN database or the empirical expression developed by Pierluisi [37] and presented in Equation (9). Alternatives to RTMs mentioned here allow the computation of the oxygen transmittance while taking into account its dependency on temperature and pressure conditions.

### 6.3 Utilizing an RTM

Lastly, case (4) (Section 4.4) follows the typical atmospheric correction scheme applied to correct airborne data and its posterior coupling with the SFM. This way, there are no mathematical inconsistencies and the convolution is performed following Equation (12). Consequently, compared to case (3), more accurate SIF estimations resulted especially outside the dip of the O_2_–A band. Following this strategy, SIF can be estimated for all the sensor heights evaluated (3–20 m) with a relative error lower than 10% across the O_2_–A absorption region when using high spectral resolution sensors (≤0.4 nm). For lower spectral resolution sensors, only SIF estimated at those wavelengths close to the bottom of the O_2_–A absorption band were within 10% of accuracy. Without a doubt, the lower spectral resolution leads to lower sensitivity for quantifying spectral differences between modelled vs. measured signals as part of the SFM. However, apart from this fact, the explanation about why in this section poorer results are achieved for lower resolution sensors can be explained by the application of the same SFM without changing any of the following stopping criteria that halt the iterative process: the threshold in the $\vec{x_{\rho }}$, $\vec{x_{F}}$ increasing steps, the threshold in the $\arg_{\vec{x_{F}},\vec{x_{\rho }}}$, or the maximum number of iterations allowed; which can be insufficient when decreasing the SR. In addition, other considerations must be highlighted in case (4) regarding the use of a RTM in order to simulate the atmospheric transfer functions involved in Equation (12). The use of an RTM implies that the final accuracy of the estimate SIF is highly dependent on the accuracy achieved on characterizing the atmospheric state.

In this paper, an ideal atmospheric characterization is assumed, showing therefore the maximum accuracy expected on the estimated SIF following this technique. As pointed out in Section 3.3, a wide number of parameters can generally be varied in atmospheric RTMs to characterize the atmospheric state. In this respect, when using the downward acquired solar irradiance at sensor level (*E*) to model the atmospheric state, this can be easily performed by minimizing the spectral error between the measured versus the modelled solar irradiance spectrum. In this respect, the separate measurement of solar irradiance terms for the global (*E*) and diffuse (*E_dif_*) contributions can improve the atmospheric characterization process since the diffuse component better accounts for aerosol scattering.

### 6.4 Other Factors Influencing SIF Retrievals

Regarding the up–scaling issues: BRDF effects, footprint variability, and scale mismatch are still factors that are difficult to characterize in linking and up–scaling remotely sensed SIF and E–C data. In this context, notable advances in UAV technology currently provide an opportunity to face these challenges [5]. While optical systems mounted on towers can monitor the vegetation canopy of interest from a fixed height (5~50 m), UAVs can attain heights of hundreds of meters, and are flexible enough to acquire the same target from different heights. However, most of the atmospheric approximations assumed for tower–basis cannot be met for UAVs. For instance, while in this work it was demonstrated that only compensating for oxygen absorption effects was enough to properly estimate SIF from a few meters distance; conversely, aerosols effects should also be compensated when processing data from UAVs. Certainly, the impact of aerosols on the estimated SIF from UAVs will definitively depend on the atmospheric conditions and the sample–sensor distance. Overall, the application of a full atmospheric correction strategy, like those applied for airborne sensors, will avoid errors in the estimated SIF caused by ignoring atmospheric effects. Apart from the distinct impact of atmospheric effects and the contrasting spatial scale, when aiming to validate satellite-derived SIF by making use of proximal sensing measurements, the different instrument’s specifications such as the spectral resolution, the signal-to-noise ratio (SNR), the level of stray-light contamination or the instrument polarization sensitivity, must also be taken into account. In terms of the SNR, since it is expected that areas selected for satellite validation will be homogeneous enough to be representative of the satellite pixels provided at a lower spatial resolution, the aggregation of multiple proximal sensing measurements can increase the effective SNR of the proximal sensing instrument, which is generally lower than the SNR of instruments on board satellites. The up-scaling process (and aspects to be taken into account) is certainly an essential field of study with strong implications on satellite validation strategies and protocols. To further investigate this issue, the Fluorescence Across Space and Time: FAST field campaign [58] has been recently dedicated to measuring SIF from leaf level to tower, UAV and satellite scale.

### 6.5 Environmental Factors Affecting SIF

Finally, this paper aims to draw attention to some aspects that are typically ignored or considered as secondary, such as the O_2_ transmittance dependency on the environmental conditions, e.g., *T* and p. This dependency, analysed in this work within a theoretical framework and following the approximation of [37], may imply the detection of trends in the retrieved SIF that are not actually related to the emitted signal but are caused by a failure to apply a proper oxygen transmittance compensation. The impact of seasonal changes in *T*,*p* was more significant for the O_2_–A than for the O_2_–B spectral region. Therefore, for proximal sensing scenarios, attention should be paid to the O_2_–A absorption lines. Results indicate that summer–winter transitions (i.e., in this case with *T* and *p* variations of ~40 *°*C and ~ 50 mbar corresponding to measurements taken at a latitude of ~60 *°*N) can produce changes in radiance units as large as 0.25 [mW/m^2^/sr/nm], which can represent a significant fraction of the SIF signal. However, implications for other regions of the planet such as the tropics do not become so critical since no abrupt *T* and *p* variations are expected in these regions during winter–summer transitions.

Although focus must be especially kept on long-temporal data series, when having the possibility of measuring a non-fluorescence target, this can provide an adequate validation strategy. The validation can be achieved by computing the reflectance spectrum at surface level by using radiance measured at the top of the tower, corrected by modelling the oxygen transmittance using the measured *T* and *p* conditions. If the measured *p* and *T* conditions are accurately estimated, no peaks should be observed in the derived surface reflectance, and therefore this test can be used as a kind of validation step. This could be the case, for instance, of rotating systems that in certain angles monitor non-vegetated (and therefore non-fluorescence) areas.

## 7 Conclusions

When estimating the weak Sun–Induced chlorophyll Fluorescence (SIF) signal by resolving the strong O_2_–A absorption region and using passive remote sensing techniques, atmospheric effects must always be compensated, even at proximal–sensing scenarios. Among all possible atmospheric effects that can impact SIF retrievals on proximal-sensing scenarios, the accurate compensation of the oxygen absorption is of most importance. In this respect, incorporating the oxygen transmittance spectral function in the formulation of the classical SIF retrieval strategies, such as the Fraunhofer Line Discriminator (FLD) or the Spectral Fitting Method (SFM) family of techniques, involves applying some algebra between non-smooth spectral functions already convolved according to the instrument resolution, which leads to unduly mathematical formulations. In this paper, we reported that compensating for oxygen transmittance with the 3FLD technique leads to a SIF estimation with relative errors between 8% (17%) and 20% (50%) for a spectral resolution (SR) of 0.1 nm (1 nm) and a target–sensor distance between 3 to 20 m. Similarly, estimated SIF at the bottom of the O_2_–A band when compensating for oxygen effects on SFM derives slightly more accurate results in all the sensor configurations evaluated, from 5% (6%) to 24% (31%) for a SR of 0.1 nm (1 nm). Hence, in order to simultaneously deal with the correction of the oxygen effects while defining a proper formulation that consistently operates the algebra at high spectral resolution, we proposed to adapt a classical airborne atmospheric correction strategy for proximal-sensing scenarios. The proposed strategy makes use of an atmospheric radiative transfer model (RTM) to characterize the target–sensor optical path. Assuming a perfect atmospheric modelling, the use of an airborne atmospheric correction scheme becomes the preferred strategy, being able to accurately estimate SIF within 10% of relative error for all the sensor spectral resolutions between 0.1 nm and 1 nm in the spectral region of 759–768 nm. Certainly, while the accuracy achieved by making use of an atmospheric RTM will be strongly dependent on the auxiliary data available to characterize the atmospheric state; no errors will be derived in this case due the mathematical formulation assumed. Finally, given the key role of the oxygen absorption effects on SIF retrievals, this work also addressed the need to take into account the dependence of oxygen absorption on air temperature (*T*) and pressure (*p*) conditions. This fact is of particular relevance for those experiments measuring long temporal SIF data series, especially at latitudes subject to strong *T* and *p* seasonality effects.

## Supplementary Material

**Supplementary Materials**: The following are available at http://www.mdpi.com/2072-4292/10/10/1551/s1.

Supplementary Material

## Abbreviations

The following abbreviations are used in this manuscript:

AOT: Aerosol Optical Thickness
EVI: Enhanced Vegetation Index
FLD: Fraunhofer Line Discriminato
FLEX: FLuorescence EXplorer
FOV: Field Of View
GOME–2: Global Ozone Monitoring Mission–2
GOSAT: Greenhouse Gases Observing Satellite
GPP: Gross Primary Productivity
HG: Henyey–Greenstein
HITRAN: HIgh–resolution TRANsmission molecular absorption database
ISRF: Instrumental Spectral Response Function
MODTRAN: MODerate TRANsmission molecular absorption database
NDVI: Normalized Difference Vegetation Index
OCO–2: Orbiting Carbon Observatory–2
RTM: Radiative Transfer Model
SFM: Spectral Fitting Methods
SIF: Solar–Induced chlorophyll Fluorescence
SNR: Signal To Noise Ratio
SPECNET: Spectral Network
SR: Spectral Resolution
SSI: Spectral Sampling Interval
SZA: Solar Zenith Angle
TOA: Top Of Atmosphere
TOC: Top Of Canopy
UAV: Unmanned Aerial Vehicle
VZA: Visual Zenith Angle
