# Supplementary material for: Compensation of Oxygen Transmittance Effects for Proximal Sensing Retrieval of Canopy–Leaving Sun-Induced Chlorophyll Fluorescence
Source: Remote Sens (Basel). Author manuscript; Available in PMC 2022 Sep 7. (PMC7613352; doi:10.3390/rs10101551)
Supplement: Supplementary Material [file EMS152624-supplement-Supplementary_Material.pdf]

Abbreviations

The following abbreviations are used in this manuscript:

AOT	Aerosol Optical Thickness
EVI	Enhanced Vegetation Index
FLD	Fraunhofer Line Discriminator
FLEX	FLuorescence EXplorer
FOV	Field Of View
GOME–2	Global Ozone Monitoring Mission–2
GOSAT	Greenhouse Gases Observing Satellite
GPP	Gross Primary Productivity
HG	Henyey–Greenstein
HITRAN	HIgh–resolution TRANsmission molecular absorption database
ISRF	Instrumental Spectral Response Function
MODTRAN	MODerate TRANsmission molecular absorption database
NDVI	Normalized Difference Vegetation Index
OCO–2	Orbiting Carbon Observatory–2
RTM	Radiative Transfer Model
SFM	Spectral Fitting Methods
SIF	Solar–Induced chlorophyll Fluorescence
SNR	Signal To Noise Ratio
SPECNET	Spectral Network
SR	Spectral Resolution
SSI	Spectral Sampling Interval
SZA	Solar Zenith Angle
TOA	Top Of Atmosphere
TOC	Top Of Canopy
UAV	Unmanned Aerial Vehicle
VZA	Visual Zenith Angle

Appendix A

This appendix summarizes in Table A1 the most relevant MODerate resolution atmospheric TRANsmission (MODTRAN) input parameters used to simulate data used in Sections 2.1 and 4. For the sake of completeness, surface reflectance and fluorescence spectra used in this work are shown in Figure A1.

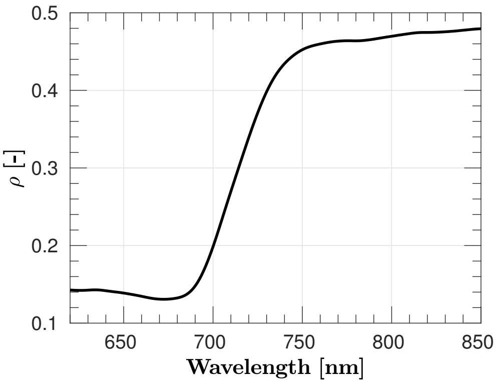

(**a**)

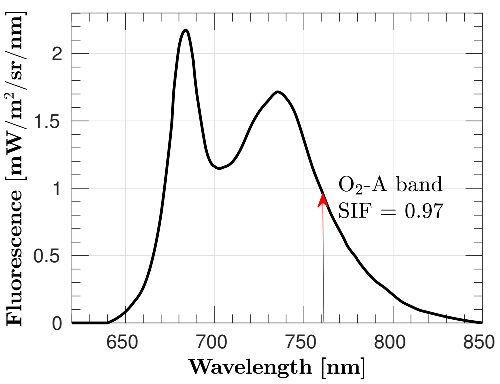

(**b**)

Figure A1. Surface reflectance (**a**) and fluorescence spectra(**b**) used in Section 4. These spectra were originally derived from the Fluorescence EXplorer (FLEX) mission requirement document [59], where they are used as a reference dataset. The red upward arrow indicates the SIF value at the bottom of the O_2–A absorption band.

Table A1. MODTRAN input parameters used to generate data from Section 2.1. MODTRAN Mid Latitude Summer (MLS) atmospheric model was selected for the sake of compatibility with numerous SIF field campaign measurements taken over Europe and United States during the spring and summer seasons. MODTRAN radiance with scattering mode was used to compute the total solar irradiance at different elevations. Solar irradiance at different elevations was computed by changing the surface elevation parameter. Total solar irradiance reaching the surface, accounting the direct and diffuse contribution, was computed by following the MODTRAN interrogation technique described in [60] and assuming a perfect reference panel having a Lambertian surface reflectance of ρ = 1. Simulations were performed using the correlated-K slow option with `DISORT` (8 streams) selected and the multiple scattering option `IMULT` activated. Highlighted geometry parameters in bold are used in simulations performed in Section 4.

	**MODTRAN Input Parameter**	**Value (Units)**
Atmospheric parameters (total column)	Model of atmosphere	Mid Latitude Summer
	AOT at 550 nm	0.15 (-)
	Aerosol Type	Rural (-)
	Water vapour	2.5 (g/cm^2)
Geometry parameters	sensor elevation	**0**, 3, 10, 20, 50 (m)
	Solar Zenith Angle	0, 20, **40**, 60 (°)
	Viewing Zenith Angle	**0** (°)
	Relative Azimuth Angle between sun and sensor	**90** (°)
High Spectral Resolution	Spectral Resolution at O_2–B	1 (cm^{-1}) ~0.04 (nm)
	Spectral Resolution at O_2–A	1 (cm^{-1}) ~0.05 (nm)

Appendix B

Air temperature (T) registered in Hyytiälä Forest Field Station in the year 2016 for a sensor height of 33 m. Air pressure (p) computed at 33 m by means of Equation (13) using air pressure registered in the year 2016 at surface level. All the data are available in https://avaa.tdata.fi/web/smart/smear.

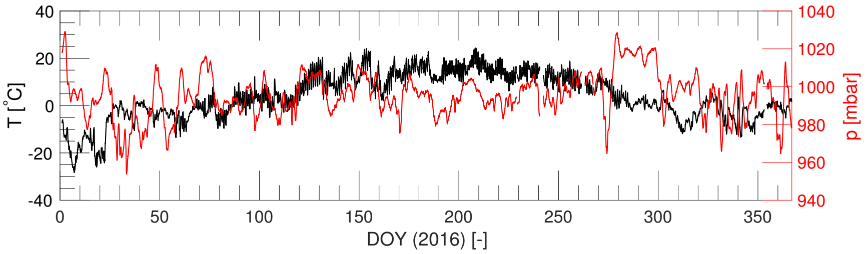

Figure A2. Annual temperature (black) and air pressure (red) registered in the Hyytiälä Forest Field Station during the year 2016.

References

1. Porcar-Castell, A.; Tyystjarvi, E.; Atherton, J.; van der Tol, C.; Flexas, J.; Pfundel, E.E.; Moreno, J.; Frankenberg, C.; Berry, J.A. Linking chlorophyll a fluorescence to photosynthesis for remote sensing applications: Mechanisms and challenges. *J. Exp. Bot.* **2014**, 1–31. [CrossRef] [PubMed]
2. Zhang, Q.; Fan, Y.; Zhang, Y.; Chou, S.; Ju, W.; Chen, J.M. A conjunct near-surface spectroscopy system for fix-angle and multi-angle continuous measurements of canopy reflectance and sun-induced chlorophyll fluorescence. In Proceedings of the SPIE Optical Engineering + Applications. International Society for Optics and Photonics, San Diego, CA, USA, 19 September 2016; p. 99770C.
3. Balzarolo, M.; Anderson, K.; Nichol, C.; Rossini, M.; Vescovo, L.; Arriga, N.; Wohlfahrt, G.; Calvet, J.C.; Carrara, A.; Cerasoli, S.; et al. Ground-based optical measurements at European flux sites: A review of methods, instruments and current controversies. *Sensors* **2011**, *11*, 7954–7981. [CrossRef] [PubMed]
